# Bacterial Cellulose Production by a Novel *Levilactobacillus brevis* Isolate Using Response Surface-Optimised Agro-Industrial Substrates

**DOI:** 10.3390/foods15020394

**Published:** 2026-01-22

**Authors:** Panyot Mongkolchat, François Malherbe, Enzo Palombo, Vito Butardo

**Affiliations:** 1 Department of Chemistry and Biotechnology, Swinburne University of Technology, Hawthorn, VIC 3122, Australia; pmongkolchat@swin.edu.au (P.M.); fmalherbe@swin.edu.au (F.M.); epalombo@swin.edu.au (E.P.); 2 Department of Science Service, Ministry of Higher Education, Science Research and Innovation, Ratchathewi, Bangkok 10400, Thailand

**Keywords:** bacterial cellulose, Box–Behnken design, circular economy, *Levilactobacillus brevis*, optimisation, response surface methodology

## Abstract

High culture medium costs economically constrain bacterial cellulose (BC) production. In parallel, agro-industrial wastes are plentiful but often underutilised sources of carbon and nitrogen substrates that could support microbial growth and metabolite production. This study aimed to bioconvert agro-industrial waste sustainably into BC using response surface methodology. A novel lactic acid bacterium, *Levilactobacillus brevis* DSS.01, isolated from nata de coco wastewater, was evaluated alongside *Acetobacter tropicalis* KBC and *Komagataeibacter xylinus* TISTR 086 for BC production using Australian agro-industrial wastes. Preliminary screening identified pear pomace and rice bran as optimal low-cost carbon and nitrogen sources, respectively. The response surface methodology employing Box–Behnken Design determined the optimal agro-industrial waste medium composition for *L. brevis* DSS.01 to produce BC at 1.56 ± 0.15 g/L. The optimised agro-industrial waste medium substituted 85% of standard Hestrin-Schramm medium components, suggesting a significant reduction in culture medium and production costs. Scanning electron microscopy revealed BC fibres from *L. brevis* DSS.01 maintained a uniform diameter. Fourier transform infrared spectroscopy and X-ray diffraction analyses indicated minimal structural deviation in BC produced from optimised agro-industrial waste medium versus standard medium. These findings demonstrate economic and sustainable BC production through valorisation of agro-industrial residues, establishing lactic acid bacteria as alternative BC producers with potential food-grade applications in circular economy frameworks.

## 1. Introduction

Bacterial cellulose (BC) is an emerging biopolymer that has garnered significant attention due to its unique properties, including high purity, high strength, and biocompatibility [1,2]. BC is commonly produced by acetic acid bacteria, such as *K. xylinus*, using a glucose-based medium [3]. However, the high cost of the synthetic culture medium is a critical barrier to the economic production of BC [4]. Agro-industrial wastes rich in carbon and nitrogen sources offer a promising, low-cost, and sustainable alternative for BC production [5]. Repurposing these nutrient-rich waste streams as feedstocks for BC fermentation proposes to offset the costs while mitigating the environmental burden of waste accumulation.

Several studies have sought to reduce production costs by replacing expensive components in synthetic culture medium with various agro-industrial wastes. These include fruit pomaces, sugarcane molasses, corn steep liquor, and rice bran, which can serve as potential carbon and nitrogen sources [2,6]. Among these options, pear pomace and rice bran are particularly noteworthy, given Australia’s significant production of both agro-industrial wastes. In Australia, apple and pear juice production is about 110,000 tons annually, resulting in over 30,000 tons of pomace or pulp by-products [7]. Additionally, Australia’s rice production for the period from 2020 to 2025 is estimated at around 545,000 tons [8], of which approximately 54,000 tons (10% of the paddy rice weight) are processed into rice bran during milling [9].

Optimising the BC production can be effectively achieved through statistical experimental design. The Box–Behnken design (BBD) is a statistical experimental design particularly valuable in response surface methodology (RSM). It is especially beneficial for exploring the relationships between multiple independent variables and the response of interest, as well as for developing mathematical models [10,11]. This approach allows for the simultaneous, systematic, and efficient variation in all parameters, in contrast to the one-factor-at-a-time (OFAT) method, which is time-consuming and expensive [12]. Previous studies have demonstrated that using RSM to optimise the medium components for *Gluconacetobacter persimmonis* resulted in a significant increase in BC yield compared to the unoptimised medium [13]. Additionally, RSM has been reported to enhance BC production from pineapple peel waste [14]. However, applying the BBD poses a challenge, as there are limited reports on optimising a medium that incorporates both cost-effective carbon and nitrogen sources to maximise BC production by lactic acid bacteria.

This study aims to investigate the effects of using agro-industrial wastes as alternative sources of carbon and nitrogen for producing BC by *L. brevis* DSS.01. The research employs a BBD and compares the results with those obtained from *A. tropicalis* KBC and *K. xylinus* TISTR 086. Additionally, the BC pellicles produced from the different cultures were analysed for their physicochemical properties. The findings highlight the potential for sustainable bioprocess optimisation using RSM to transform waste into high-value biomaterials, address the cost limitations associated with BC’s culture medium, and mark a significant advancement in green biotechnology with broad implications for industry.

## 2. Materials and Methods

### 2.1. Cellulose-Producing Bacteria, Chemicals and Reagents, and Agro-Industrial Waste Residues

The strain *L. brevis* DSS.01 was isolated from nata de coco wastewater, and *A. tropicalis* KBC was isolated from kombucha tea. Both isolates were identified through biochemical analysis and 16S ribosomal RNA gene sequencing (Supplementary Tables S1 and S2; Supplementary Figures S1 and S2). The reference strain *K. xylinus* TISTR 086 was purchased from the Thailand Institute of Scientific and Technological Research (TISTR) Culture Collection, Pathum Thani, Thailand. D-glucose, peptone, yeast extract, disodium hydrogen phosphate, citric acid, magnesium sulfate heptahydrate, sodium hydroxide, alternative carbon sources, and nitrogen sources were ordered from Thermo Fisher Scientific Australia Pty Ltd., Scoresby, VIC, Australia and Chem-Supply Pty Ltd., Gillman, SA, Australia. The agro-industrial wastes, including apple pomace (AP), beetroot pomace (BP), and pear pomace (PP), were supported by the Australian Fruit Solution, Shepparton, VIC, Australia. Stabilised rice bran (RB) was provided by SunRice, Leeton, NSW, Australia, and waste beer slurry (WBS) was obtained from the Inner North Brewing Company, Brunswick, VIC, Australia. All agro-industrial wastes were oven-dried at 50 °C until constant weight was reached. Then, the dried waste was ground and sieved through a 0.5 mm mesh sieve. The homogeneous powder with particle size less than 0.5 mm was stored in a secure plastic container at room temperature.

### 2.2. Inoculum Preparation

A glycerol stock of *L. brevis* DSS.01, *A. tropicalis* KBC, and *K. xylinus* TISTR 086 was inoculated into 50 mL centrifuge tubes containing 30 mL of Hestrin-Schramm (HS) medium, which consists of the following components (expressed as % *w*/*v*): 2.0% D-glucose, 0.5% yeast extract, 0.5% peptone, 0.27% Na_2_HPO_4_, and 0.115% citric acid, with pH 6. The cultures were then incubated at 28 °C for 14 days under static conditions. After that, the incubated culture was normalised to an Optical Density (O.D.) of 0.1 at 600 nm. Then, 10% (*v*/*v*) of the normalised inoculum was subcultured in HS medium to increase the volume to 400 mL in 500 mL Duran bottles, and the bottles were incubated under the same conditions. The prepared inoculum was checked for purity and normalised by the O.D. prior to use in further experiments, which were all conducted in 50 mL centrifuge tubes containing 27 mL of medium.

### 2.3. Preliminary Optimisation of Culture Medium Using the One-Factor-at-a-Time Approach

The influence of variables, including BC-producing strains, carbon sources, nitrogen sources, and MgSO_4_, was primarily investigated through the OFAT method. Briefly, 10% (*v*/*v*) of normalised inoculum, *L. brevis* DSS.01, *A. tropicalis* KBC, and *K. xylinus* TISTR 086, were individually inoculated into HS medium, pH 6, to assess the influence of BC producers. To observe the effect of the carbon source, glucose in the HS medium was replaced with fructose, lactose, sucrose, mannitol, glycerol, and starch. Meanwhile, a mixture of yeast extract and peptone in HS medium was replaced by organic nitrogen sources (yeast extract and peptone) and inorganic nitrogen sources (ammonium acetate, ammonium chloride, ammonium nitrate, ammonium sulfate, and casamino acid) to investigate the effect of nitrogen sources. The significance of a variable in BC production was observed by inoculating 10% (*v*/*v*) of normalised inoculum into HS medium with different concentrations of glucose (0, 1, 2, 3, 4% *w*/*v*), yeast extract (0, 0.5, 1.0, 1.5, and 2.0% *w*/*v*), and MgSO_4_ (0, 0.2, 0.4, 0.6, 0.8% *w*/*v*). All samples were then incubated at 28 °C under static conditions. After 14 days of cultivation, the BC was harvested, purified, and the BC production yield was measured. All experiments were fixed at an incubation time of 14 days to ensure consistency in BC thickness and quality across samples. Based on the preliminary investigation, shorter incubation times resulted in insufficient BC pellicle formation for reliable measurement.

### 2.4. Replacing Conventional Medium Ingredients with Agro-Industrial Wastes

#### 2.4.1. Screening of Agro-Industrial Waste for Culture Medium Optimisation

Agro-industrial wastes for optimisation were primarily selected by directed substitution of carbon and nitrogen in the HS medium with cost-effective carbon sources (AP, BP, and PP) and nitrogen sources (RB and WBS) at the same weight of glucose, peptone, and yeast extract in the HS medium at pH 6 (Supplementary Table S3). The 10% (*v*/*v*) of normalised inoculum was inoculated and then incubated at 28 °C for 14 days under static conditions. Agro-industrial waste that provided a high BC production was selected and sent to Eurofins Scientific (Dandenong South, VIC, Australia) for proximate chemical composition analysis.

#### 2.4.2. Response Surface Methodology Approach for Culture Medium Optimisation

RSM was applied to evaluate and optimise the combined effects of three independent variables on BC production. Among the available RSMs, the BBD was chosen because it efficiently estimates quadratic response surfaces while requiring fewer experimental runs than a full factorial design. BBD is particularly suitable for biological systems because it avoids treatment combinations in which all factors simultaneously reach extreme levels, thereby reducing the risk of experimental failure [11].

In this study, the independent variables, pear pomace (PP), rice bran (RB), and MgSO_4_ were actually coded as *A*, *B*, and *C*, respectively. Each actual code was applied at three experimental levels (−1, 0, and +1), representing low, centre, and high. The design generated a set of experimental combinations that allowed estimation of linear, interaction, and quadratic effects [11,12]. The actual code and experimental levels used for each factor are shown in Table 1. A total of 17 runs, comprising 12 full factorial BBD runs and five central runs, were utilised to observe the individual and synergistic effects of the three independent variables.

The experimental results were fitted to the following second-order polynomial structured model for three variables using Equation (1).

*Y* = *β*_0_ + *β*_1_(*A*) + *β*_2_(*B*) + *β*_3_(*C*) + *β*_12_(*AB*) + *β*_13_(*AC*) + *β*_23_(*BC*) + *β*_11_(*A*)^2^ + *β*_22_(*B*)^2^ + *β*_33_(*C*)^2^, (1)

where *Y* is the predicted response; *A*, *B*, and *C* are the actual codes of independent variables (PP, RB, and MgSO_4_, respectively); *β*_0_ is the intercept; *β*_1_, *β*_2_, and *β*_3_ are linear regression coefficients; *β*_12_, *β*_13_, and *β*_23_ are interaction regression coefficients, and *β*_11_, *β*_22_, and *β*_33_ are square regression coefficients.

### 2.5. Purification and Quantification of Bacterial Cellulose

After cultivation, BC pellicles were harvested from the culture medium and rinsed thoroughly with distilled water to remove residual medium components. The pellicles were then treated with 0.1 M NaOH at 80 °C for 60 min to remove bacterial cells and medium residues. The pellicles were then neutralised with distilled water and dried in an oven at 60 °C until a constant weight was achieved. Finally, BC production and water content were calculated using Equations (2) and (3).

BC production (g/L) = *W*_dry_ (g)/Volume of culture medium (L), (2)

Water content (%) = [*W*_wet_ (g) − *W*_dry_ (g)/*W*_wet_ (g)] × 100, (3)

where *W*_dry_ is the dry weight, and *W*_wet_ is the wet weight of BC.

### 2.6. Comprehensive Characterisation of Bacterial Cellulose Materials

#### 2.6.1. Surface Morphology Analysis

The surface morphology was determined by using scanning electron microscopy (SEM) (Zeiss SUPRA-40, Oberkochen, Germany). The dried BC pellicles were initially mounted on a stub and coated with gold. The morphology was observed using SEM at an accelerating voltage of 15 kV and a magnification of 20,000× to determine the surface structure of BC. The fibre diameter was measured from 100 replicated measurements using ImageJ software (ImageJ version 1.53k, National Institutes of Health, Bethesda, MA, USA).

#### 2.6.2. Fourier Transform Infrared Spectroscopy (FTIR) Analysis

FTIR analysis was conducted using FTIR Vertex 70 (Bruker, Karlsruhe, Germany). The spectrophotometric transmittance mode, FTIR data were recorded at 16 cm^−1^ resolution over the wavelength range 400–4000 cm^−1^ with an average of 200 scans for thin BC pellicles. The dried BC pellicles were mounted directly in the sample compartment, as samples were sufficiently thin to allow IR transmission. Spectra were acquired in absorbance mode and, where required, converted to transmittance using OPUS software version 7.5 (Bruker Optics, Ettlingen, Germany). Then, the spectrum line was baseline-corrected and normalised on the generated IR spectra using Quasar 1.7.0 (Canadian Light Source (CLS), Saskatchewan, SK, Canada) and Origin 2023 statistical software (OriginLab Corporation, MA, USA).

#### 2.6.3. X-Ray Diffraction (XRD) Analysis

The change in crystallinity of dried BC pellicles was evaluated using an X-ray diffractometer (XRD D8, Bruker, Karlsruhe, Germany) equipped with Cu Kα radiation at 40 kV and 25 mA. Data were collected in reflection mode with a 5–80° (2θ) scanning speed of 0.5 s/step, 0 rpm, a 10 × 10 mm variable slit, and automatic air scatter. The Crystallinity Index (CI) was calculated using Equation (4).

Crystallinity Index (CI) = (*I*_002_ − *I*_am_)/*I*_002_ × 100 (4)

where *I*_002_ is the highest intensity above the baseline at 2θ, which is approximately 22.5°, corresponding to the crystalline region, and *I*_am_ is the minimum peak located between the (110) and (200) peaks, which is around 18°, corresponding to the amorphous region.

### 2.7. Cost Evaluation of Culture Medium Inputs

The cost of agro-industrial waste and the standard HS medium were compared, and the simplified percentage cost savings were reported based on the culture medium input. The costs of the medium components were obtained from supplier price lists.

### 2.8. Statistical Analysis

All experiments were performed in triplicate except for the proximate chemical composition analysis of PP and RB. The data were expressed as mean ± standard deviation. Significant differences were determined according to analysis of variance (ANOVA) by Tukey’s multiple comparisons test at *p* ≤ 0.05 using Origin 2023 statistical software (OriginLab Corporation, Northampton, MA, USA). The BBD data analysis, which involved developing regression models and plotting three-dimensional surface plots, was conducted using multiple regression in Design-Expert (Version 13, Stat-Ease, Minneapolis, MN, USA).

## 3. Results

### 3.1. Pre-Optimisation of Culture Medium Using the One-Factor-at-a-Time Method

#### 3.1.1. Effect of BC-Producing Strains on Bacterial Cellulose Production

**Figure 1:**
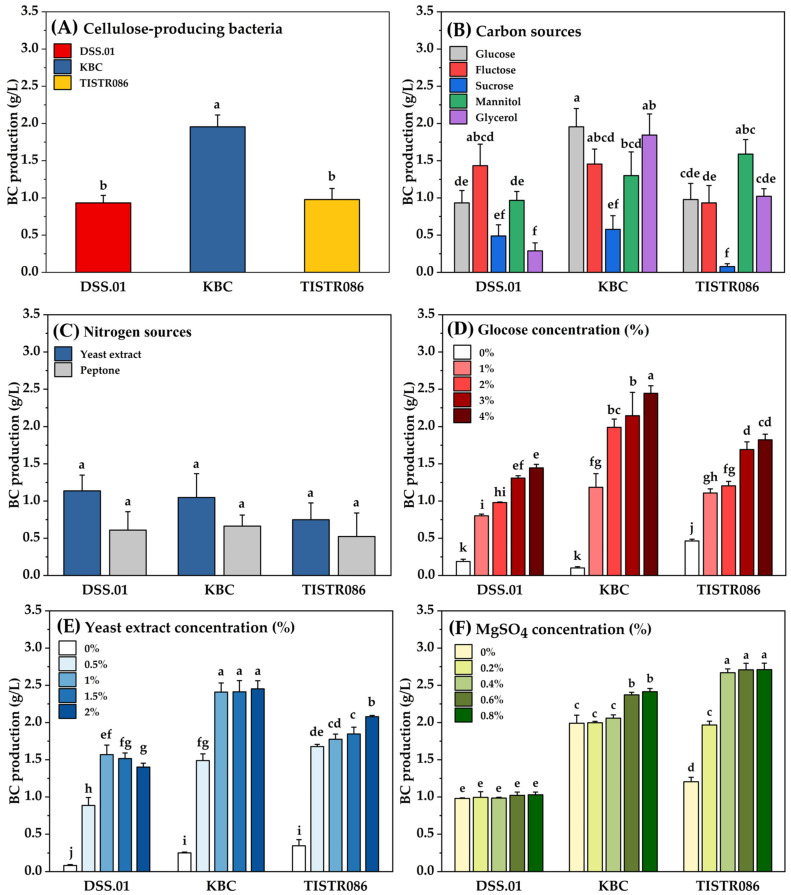
The effect of different variables on BC production: (**A**) cellulose-producing bacteria, (**B**) carbon sources, (**C**) nitrogen sources, (**D**) glucose concentration, (**E**) yeast extract concentration, and (**F**) MgSO_4_ concentration. Different small letters (a–k) indicate statistically significant differences among treatments based on post-hoc multiple comparison tests (*p* < 0.05). Treatments sharing the same letter are not significantly different.

The BC production of *L. brevis* DSS.01 (0.93 ± 0.17 g/L) was comparable to that obtained from *K. xylinus* TISTR 086 (0.97 ± 0.22 g/L). Among the three BC producers, *A. tropicalis* KBC showed the highest BC production (1.96 ± 0.25 g/L) in the standard HS medium (Figure 1A), proving that a diverse group of microorganisms can synthesise BC. The isolated *L. brevis* DSS.01 is a specific strain within the *Lactobacillus* genus that may possess cellulose synthase-like operons in its genes, requiring further bioinformatic validation due to the lack of previous literature reporting cellulose synthase operons in these LAB. Previous studies suggested that *L. brevis* contains encoded genes that enable bacteria to adhere to cellulose and Xylan [15]. Adetunji & Adegoke (2007) reported that *L. brevis* can produce BC without comprehensive characterisation [16].

#### 3.1.2. Effect of Carbon Sources on Bacterial Cellulose Production

Varying carbon sources in the culture medium revealed distinct metabolic preferences among the tested cellulose-producing bacteria, each favouring different substrates to optimise cellulose biosynthesis (Figure 1B). Among the evaluated carbon sources, fructose, mannitol, and glucose emerged as superior options. The isolated *L. brevis* DSS.01 produced the highest BC production with fructose (1.43 ± 0.29 g/L), followed by mannitol (0.97 ± 0.12 g/L) and glucose (0.93 ± 0.17 g/L). In contrast, *A. tropicalis* KBC exhibited its highest BC production with glucose (1.96 ± 0.25 g/L), glycerol (1.84 ± 0.28 g/L), and fructose (1.46 ± 0.2 g/L). At the same time, *K. xylinus* TISTR 086 showed a preference for mannitol (1.59 ± 0.2 g/L), glycerol (1.02 ± 0.1 g/L), and glucose (0.98 ± 0.22 g/L). Interestingly, all three BC producers cannot use lactose and starch for BC biosynthesis. Starch and lactose cannot be effectively used as primary carbon sources for BC because cellulose-producing bacteria typically lack the specific enzymes required to break down these complex sugars into the simple monosaccharides [17], even though lactose is an essential carbon source for LAB’s growth and lactic acid production [18]. No previous study reported the use of lactose for BC production by cellulose-producing LAB.

#### 3.1.3. Effect of Nitrogen Sources on Bacterial Cellulose Production

Nitrogen sources play an indirect role in BC production by supporting microbial growth, enzyme synthesis, and metabolic activities [19]. In this study, none of the bacterial strains produced BC when cultivated in a medium containing inorganic nitrogen (Figure 1C). Inorganic nitrogen sources can cause toxicity and increase ionic strength, thereby affecting BC productivity [19]. When replacing nitrogen sources in HS medium with organic nitrogen sources, all three tested strains produced higher BC in yeast extract than those obtained from peptone: *L. brevis* DSS.01 (1.14 ± 0.21 and 0.61 ± 0.25 g/L), *A. tropicalis* KBC (1.05 ± 0.32 and 0.66 ± 0.15 g/L), and *K. xylinus* TISTR 086 (0.75 ± 0.23 and 0.52 ± 0.32 g/L).

#### 3.1.4. Effect of Glucose Concentration on Bacterial Cellulose Production

BC production showed a positive relationship with glucose concentration, with higher glucose levels resulting in increased BC yield across all three strains (Figure 1D). The results aligned with previous studies. When glucose concentrations reached approximately 10 g/L, the symbiotic culture of bacteria and yeast (SCOBY) in kombucha exhibited a marked increase in BC production [20]. The concentration of 4% glucose yields the best results for *G. hansenii* UAC09 [21]. Glucose is the primary carbon source directly utilised in cellulose biosynthesis. Moreover, glucose unit is a primary component of the BC structure, joined together via β-1,4-glycosidic linkages [22]. Excessive glucose may lead to osmotic stress, acid accumulation, or catabolite repression, which can inhibit BC biosynthesis [21].

#### 3.1.5. Effect of Yeast Extract Concentration on Bacterial Cellulose Production

BC production of *A. tropicalis* KBC and *K. xylinus* TISTR 086 increased with higher concentrations of yeast extract. The isolated *L. brevis* DSS.01 also responded positively to yeast extract; however, increasing the concentration beyond 1% resulted in a slight decrease in BC production (Figure 1E). High yeast extract concentration can lead to excess nitrogen availability and inhibit key enzymes. Thereby shifting bacterial metabolism toward biomass growth rather than secondary metabolite production, such as BC [23,24].

#### 3.1.6. Effect of MgSO_4_ on Bacterial Cellulose Production

The effect of MgSO_4_ concentration on BC production across the three strains was observed; the isolated *L. brevis* DSS.01 has a minimal response to MgSO_4_, while it had a strong positive correlation between MgSO_4_ concentration and BC production from *A. tropicalis* KBC and *K. xylinus* TISTR 086 (Figure 1F). The results aligned with previous studies. *A. xylinum* produced maximum BC thickness on culture medium supplemented with 0.12% MgSO_4_ [25]. Owing to MgSO_4_ supply magnesium ions (Mg^2+^) that enhance enzymatic activity, cell stability, and metabolic efficiency [26].

### 3.2. Culture Medium Substitution by Agro-Industrial Wastes

#### 3.2.1. Selection of Agro-Industrial Wastes for Culture Medium Optimisation

**Figure 2:**
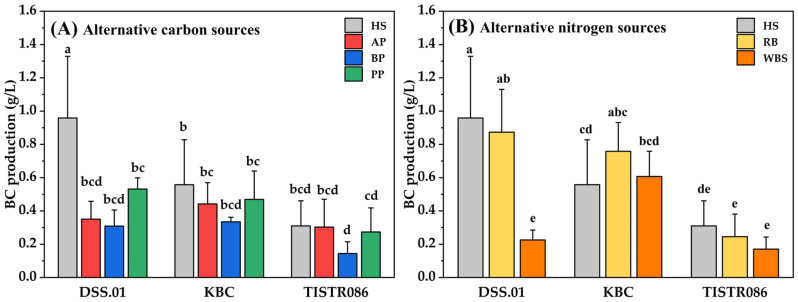
The BC production of different agro-industrial waste medium: (**A**) alternative carbon sources and (**B**) alternative nitrogen sources. Different small letters (a–e) indicate statistically significant differences among treatments based on post-hoc multiple comparison tests (*p* < 0.05). Treatments sharing the same letter are not significantly different.

When glucose in the HS medium was replaced with agro-industrial wastes as alternative carbon sources, there was no statistically significant difference observed in the BC production of all strains in AP and PP, as presented in Figure 2A. Among the three fruit wastes, PP appeared to be the most promising alternative carbon source for *L. brevis* DSS.01, with a BC production of 0.53 ± 0.07 g/L, compared with AP (0.35 ± 0.11 g/L) and BP (0.31 ± 0.10 g/L). *A. tropicalis* KBC presented a similar preference for PP (0.47 ± 0.17 g/L), AP (0.44 ± 0.13 g/L), and BP (0.33 ± 0.03 g/L). In contrast, *K. xylinus* TISTR 086 produced better BC in AP (0.30 ± 0.17 g/L) than PP (0.27 ± 0.14 g/L) and BP (0.14 ± 0.07 g/L). The comparison of BC production among cellulose-producing bacteria, *L. brevis* DSS.01 and *A. tropicalis* KBC, showed that they potentially produce BC from alternative carbon sources compared to *K. xylinus* TISTR 086. Some cellulose-producing bacteria ineffectively produce BC due to a lack of essential enzymes or the presence of inhibitory compounds in the waste medium [1,27].

The investigation of alternative nitrogen sources revealed that RB tended to be a greater substituent than WBS, as *L. brevis* DSS.01, *A. tropicalis* KBC, and *K. xylinus* TISTR 086 produced a maximum BC in RB, 0.87 ± 0.26 g/L, 0.76 ± 0.17 g/L, and 0.25 ± 0.14 g/L, respectively. It was 3.78, 1.25, and 1.47 times higher than those obtained in WBS. Furthermore, the BC production of *L. brevis* DSS.01 in RB was statistically comparable to that obtained from HS medium (*p* > 0.05) (Figure 2B). The BC yield showed a positive correlation with the quantity of RB in the culture medium. The BC yield of *G. xylinum* was 100% increased when the proportion of RB in D-mannitol: RB medium increased from 1:1 to 1:3 [28]. However, directly replacing the medium with untreated RB did not lead to a notable increase in BC yield by *Novacetimonas* sp. [22]. WBS was not suitable for BC production in this study, possibly because phenolic compounds and antimicrobial agents remaining in brewery waste can inhibit bacterial growth or enzyme activity, thereby reducing cellulose synthesis [24,27]. The findings showed that PP and RB were suitably selected for optimisation of the culture medium.

#### 3.2.2. Proximate Chemical Composition Analysis of Selected Agro-Industrial Wastes

The proximate chemical analysis revealed that PP consisted of carbohydrates (92.6%), protein (3.3%), fat (0.8%), moisture (1.3%), ash (2.0%), and magnesium (0.06%). RB comprised carbohydrates (46.9%), protein (14.7%), fat (22.4%), moisture (6.2%), ash (9.8%), and magnesium (0.98%).

Previous studies described that the carbohydrate composition of PP is predominantly characterised by complex, polymeric carbohydrates, including a significant proportion of 89.2% insoluble dietary fibre (cellulose, hemicellulose, and lignin), 1.5% soluble dietary fibre (pectin and β-glucan), and 0.3% free sugars and sugar alcohols (fructose, glucose, sorbitol, and sucrose) [29,30]. Similarly, a major chemical composition of RB is complex and polymeric carbohydrates; the main carbohydrate components include insoluble starch, dietary fibre (cellulose, hemicellulose, and arabinoxylans, and lignin), soluble dietary fibre (pectin and β-glucan), and a small amount of free sugars [31]. The point is BC-producing strains typically lack amylase, cellulase, and pectinase enzymes required to hydrolyse these polymeric carbohydrates [32]. These also explained the absence of BC production when starch was supplied as a carbon source substitute.

In terms of nitrogen substitution, RB is a good source of protein, containing several essential amino acids. Stabilised RB was used in this study, and the heat treatment during culture medium preparation partially denatured and softened RB, potentially increasing the availability of soluble nutrients and trace sugars. Although heat treatment does not depolymerise the significant carbohydrate fractions (starch, cellulose, and hemicellulose), the denaturation of proteins and release of soluble nitrogen support microbial growth [33,34]. Polyphenols and other antioxidant compounds in agro-industrial wastes are significant; the limitation is that this study did not quantify them, which can influence microbial growth and lead to variations in BC production [35].

Despite numerous reports recommending agro-industrial waste hydrolysis prior to fermentation [14,36]. This study focused on using PP and stabilised RB, without hydrolysis or chemical pretreatment, as alternative carbon and nitrogen sources for BC production. This approach was chosen to reduce processing costs, simplify the production workflow, and demonstrate the effectiveness of the statistical optimisation model in enhancing BC yield under industry-relevant, minimally processed conditions.

### 3.3. Optimisation of Culture Medium Using Box–Behnken Design

The BBD results revealed that the variation in actual BC production ranged from 0.14 ± 0.03 to 1.64 ± 0.08 g/L (Table 2). Regarding the maximum BC production, each cellulose-producing bacterium has a different preferred ratio of PP, RB, and MgSO_4_. The highest BC production of *L. brevis* DSS.01, followed by *A. tropicalis* KBC, and *K. xylinus* TISTR 086, was 1.64 ± 0.08 g/L, 1.48 ± 0.02 g/L, and 0.36 ± 0.05 g/L, respectively.

The variance and regression analysis results indicated that the response (BC production) of each cellulose-producing bacterium relied on different sets of independent variables (Table 3). Overall, the model F-values of 71.87, 29.76, and 15.89 for *L. brevis* DSS.01, *A. tropicalis* KBC, and *K. xylinus* TISTR 086, respectively, indicated that all three models were significant.

Moreover, *p*-values for the model terms showed that, across the three cellulose-producing bacteria, only the individual independent variables (A, B, and C) had significant effects (*p* < 0.05). At the same time, the interaction terms (AB, AC, and BC) were non-significant (*p* > 0.1), indicating no synergistic effect between the independent variables on BC production. As the literature describes, model F and *p*-values are used to determine the model’s availability for predicting responses. Model term values of *p*-value less than 0.05 are considered significant, and values greater than 0.1 are non-significant model terms. A non-significant lack of fit indicated that the fitted quadratic models adequately represented the experimental data (*p* > 0.05) [37]. Therefore, the model can be used to predict BC production of BC-producers used in this study.

For *L. brevis* DSS.01, the R^2^ value of 0.9895 and the adjusted R^2^ of 0.9760 suggested an acceptable range. The R^2^ value close to 1.0 indicated that the model’s accuracy for predicting the response. The Adjusted R^2^ is used to calibrate R^2^ values with respect to sample size and the number of variables in the model. Adjusted R^2^ should be close to the R^2^ value [13,14]. The adequate precision of 28.37, surpassing the threshold of 4, indicates that this model is appropriate for exploring the design space [37]. The strain is highly responsive to PP concentration, with RB and MgSO_4_ also contributing significantly to BC formation. Quadratic terms (A^2^, B^2^) suggested non-linear effects, possibly optimal ranges for PP and RB. However, non-significant interactions of variables confirmed that the additive effects dominate.

For *A. tropicalis* KBC, the BC production of the actual and predicted values was well fitted (R^2^ of 0.9742 and adjusted R^2^ of 0.9411), and the model was reliable (adequate precision of 16.49). The strain *A. tropicalis* KBC was susceptible to PP and RB, but MgSO_4_ had no significant effect. The significance of B^2^ and C^2^ suggests curvature in RB and MgSO_4_ effects—possibly thresholds of RB and MgSO_4_ beyond 68.0 g/L and 0.5 g/L, at which BC production declines. Similar to *L. brevis* DSS.01, the interaction between variables was minimal.

For *K. xylinus* TISTR 086, the model was accurate (R^2^ of 0.9533 and Adjusted R^2^ of 0.8933) and reliable (Adequate Precision of 12.5485) to predict BC production. The *p*-values of model terms indicated that the BC production responded significantly to PP and RB, with strong quadratic effects. Similar to *L. brevis* DSS.01 and *A. tropicalis* KBC, there was no synergistic effect between PP, RB, and MgSO_4_ on enhancing BC production. All three models’ lack of fit was non-significant, confirming that the quadratic model adequately described the relationship between the medium components and BC yield [13].

**Figure 3:**
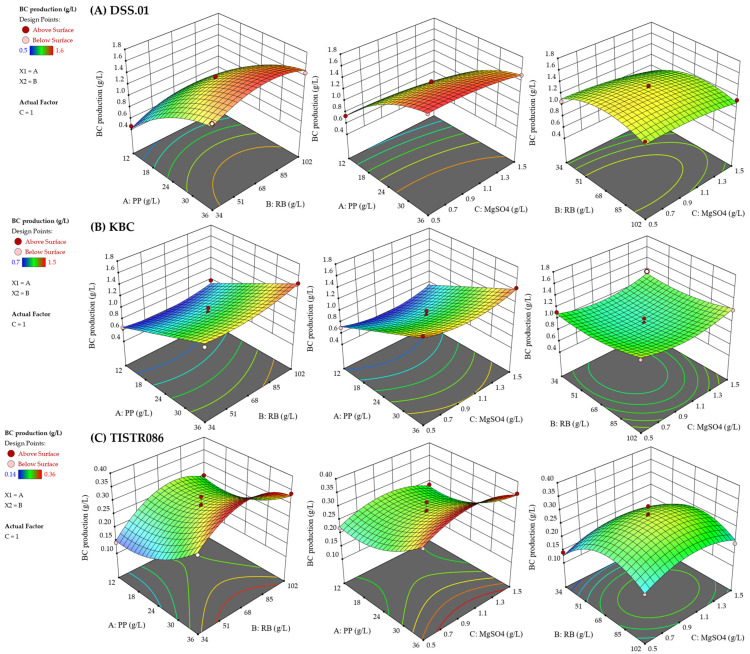
Three-dimensional surface plots representing the effect of PP, RB, and MgSO_4_ on the BC production: (**A**) *L. brevis* DSS.01, (**B**) *A. tropicalis* KBC, (**C**) *K. xylinus* TISTR 086. Colour gradient represents the predicted response surface; contour lines indicate response levels; red and pink points represent experimental values above and below the surface, respectively.

In Figure 3A, the three-dimensional surface plots of *L. brevis* DSS.01 showed a pronounced curvature of the PP concentration, as evidenced in Figure 3A, which indicates that the carbon source is a critical parameter influencing BC production. The result aligned with the pre-optimisation that *L. brevis* DSS.01 favoured in using fructose for BC production, and a predominant sugar in fruit pomace is fructose [29,30]. Therefore, *L. brevis* DSS.01 produced higher BC in the optimised agro-industrial waste medium. The three-dimensional surface plots visualised the ANOVA analysis, where PP had the highest F-value and a significant quadratic effect. On the contrary, RB and MgSO_4_ had a moderate individual effect as presented by the relatively flat surface.

The surface plots of *A. tropicalis* KBC and *K. xylinus* TISTR 086 showed the independent effect of each variable on the BC production (Figure 3B,C). There were no significant interactions between variables on BC production. The increase in BC yield was observed when presenting moderate PP and low-to-mid RB concentrations. Increasing MgSO_4_ beyond 1.0 g/L leads to a decrease in BC yield, even with optimal PP.

Additionally, BC yield dropped sharply when both RB and MgSO_4_ were present at high levels. It could be associated with the characteristic of culture medium containing a high concentration of RB, as the dispersion of RB residues made the medium turbid over time. These dispersed RB residues may interfere with BC fabrication, and some have become trapped within the cellulose pellicle, reducing BC production. Moreover, the high fibre content in RB may impede the fermentation process by affecting the solubility and availability of sugars for bacterial consumption [38]. Another possible reason is phenolic compounds, flavonoids, and γ-oryzanol of RB, which are linked to its antimicrobial properties and interfere with the growth of BC producers [39]. Excessive MgSO_4_ contributed to osmotic stress, affecting BC production [40,41].

### 3.4. Model Validation

Validation was carried out using only the predicted optimal conditions for *L. brevis* DSS.01 because this strain represents the principal novel finding of this study: a lactic acid bacterium capable of BC production, while *A. tropicalis* KBC and *K. xylinus* TISTR 086 are acetic acid bacteria with well-documented BC biosynthesis capabilities. The demonstration of BC production by a LAB using agro-industrial waste substrates constitutes the primary contribution of this work. Additionally, the substantial quantity of RB required for validation necessitated prioritisation of *L. brevis* DSS.01 for upscale production trials. The optimised agro-industrial waste medium composition (expressed as % *w*/*v*) was 3.6% PP, 6.8% RB, 0.27% Na_2_HPO_4_, 0.115% citric acid, and 0.05% MgSO_4_ at pH 6. The cultures were then incubated at 28 °C under static conditions for 14 days. There was no statistically significant difference in BC production between predicted (1.6 g/L) and actual experimental values (1.56 ± 0.15 g/L). The percentage error between predicted and actual means was 2.37%, indicating that the model’s output remains acceptably close to the experimental means. This low error supports the model’s utility for preliminary estimation, though further refinement is needed to improve predictive robustness.

After model fitting and elimination of non-significant terms of the second-order quadratic model, as presented in Equation (1). The final regression equation for BC production by *L. brevis* DSS.01 was shown in Equation (5):

BC production (DSS.01) = 1.33 + 0.47*A* + 0.077*B* − 0.083*C* − 0.17*A*^2^ − 0.2*B*^2^ (5)

where *A*, *B*, and *C* represent the actual code of PP, RB, and MgSO_4_, respectively.

This reduced quadratic model indicates that BC production was mainly governed by the linear effects of A, B, and C, together with significant curvature effects in *A* and *B*. The positive coefficients for A and B suggested that increasing PP and RB initially enhanced BC production. In contrast, the negative quadratic terms for A^2^ and B^2^ implied the presence of an optimal region beyond which further increases in BC production decline. The small negative coefficient for C indicates a slight inhibitory effect of MgSO_4_ within the experimental range. The BC pellicles harvested from the upscale production in a 3 L glass tray containing 2 L of an optimised agro-industrial waste medium are shown in Supplementary Figure S5.

### 3.5. Characterisation of Bacterial Cellulose

#### 3.5.1. Water Content

BC exhibited a high moisture content, ranging from 92.26% to 99.94%, which typically indicates enhanced water retention capacity (Supplementary Table S5).

#### 3.5.2. Surface Morphological Analysis

**Figure 4:**
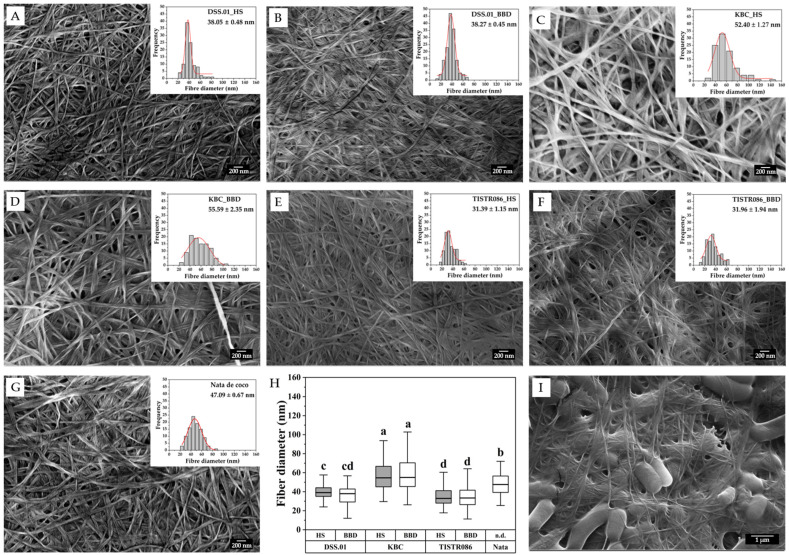
SEM micrographs and fibre size distribution of BC: (**A**) DSS.01_HS, (**B**) DSS.01_BBD, (**C**) KBC_HS, (**D**) KBC_BBD, (**E**) TISTR 086_HS, (**F**) TISTR 086_BBD, (**G**) nata de coco, (**H**) box-plot of fibre diameters, and (**I**) cellulose-producing bacterium, *L. brevis* DSS.01. Different small letters (a–d) indicate statistically significant differences among samples based on post-hoc multiple comparison tests (*p* < 0.05). Treatments sharing the same letter are not significantly different.

SEM micrographs revealed the ultrafine structure of BC produced by different cellulose-producing bacteria (Figure 4). All BC samples exhibited a three-dimensional network of interwoven nanofibres, characteristic of BC. However, notable differences in fibre dimensions were observed among the three tested cellulose-producing bacteria. *A. tropicalis* KBC produced the thickest BC fibre diameter, followed by *L. brevis* DSS.01, and *K. xylinus* TISTR 086 secreted the finest fibres. When comparing the fibre diameter of BC, which was cultivated in HS medium and optimised medium, the average fibre diameter of BC from *L. brevis* DSS.01 in HS medium and optimised medium was in the same range of 38.05 ± 0.48 nm and 38.27 ± 0.45 nm, significantly smaller than that of *A. tropicalis* KBC in HS medium and optimised medium 52.40 ± 1.27 nm and 55.59 ± 2.35 nm, respectively. In comparison, nata de coco (commercial BC) has a fibre diameter of 47.09 ± 0.67 nm. This suggests that the culture medium substitution with PP and RB may have compensated for any limiting factors, enabling the bacteria to produce cellulose with fibre diameters similar to those in the optimal HS medium (Figure 4A–H). Moreover, BC from *L. brevis* DSS.01 in HS medium exhibited very low polydispersity, indicating that most fibres were similar in size and highly uniform. Figure 4I showed *L. brevis* DSS.01, which was covered with its secreted cellulose fibres.

#### 3.5.3. FTIR Characterisation of Chemical Functional Groups

**Figure 5:**
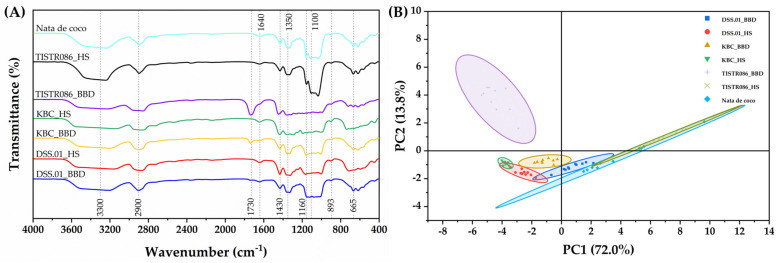
Comparison analysis of (**A**) FTIR spectra of BC and (**B**) PCA of FTIR spectra.

FTIR spectroscopy was employed to analyse the chemical composition and bonding patterns of the BC samples (Figure 5A). All samples exhibited characteristic absorption peaks associated with cellulose I structure. The spectra of BC from *L. brevis* DSS.01, *A. tropicalis* KBC, and *K. xylinus* TISTR 086 in HS medium showed similar patterns but slightly differed in peak intensity, confirming their cellulosic nature. Key absorption peaks were observed at ~3300 cm^−1^ (O-H stretching in cellulose), ~2900 cm^−1^ (C-H stretching of CH_2_ and CH_3_ groups), ~1640 cm^−1^ (H-O-H bending of absorbed water), ~1430 cm^−1^, ~1350 cm^−1^, and ~1110–1160 cm^−1^ (CH_2_ scissoring, C-H bending, and C-O-C bond of carbohydrate, respectively), ~893 cm^−1^ (β-1,4 linkage bending), ~665 cm^−1^ (O-H out-of-plane bending), aligned with previous study [22,42]. Notably, the presence of the absorption peak at ~1730 cm^−1^ of BC from three strains in an optimised agro-industrial waste medium corresponds to unconjugated C=O stretching and nearby carbonyl-related peaks, which may indicate esterified or oxidised functionalities within lignin [43]. It could be due to the RB residue dispersion, which may be stuck within the BC network formation and present a spectrum at ~1730 cm^−1^, especially in the TISTR086_BBD sample. Pretreatment of RB through solid-state extraction could be applied to enhance the RB utilisation for cellulose production [22].

The PCA of the FTIR spectra in the 400–4000 cm^−1^ region provided further insights into the similarities and differences between the BC samples (Figure 5B). The first principal component (PC1) explained 72.0% of the spectral variance, while PC2 accounted for 13.8%, collectively representing 85.8% of the total variance. The PCA score plot revealed close clustering of BC spectra of DSS.01_BBD and DSS.01_HS, which suggested minimal structural deviation between optimised agro-industrial waste and HS medium for *L. brevis* DSS.01. The separation of BC spectra of TISTR086_BBD clusters indicated that *K. xylinus* TISTR 086 is sensitive to agro-industrial waste; BC fabrication may interfere with the waste residues.

#### 3.5.4. Crystallographic Analysis Using X-Ray Diffraction

**Figure 6:**
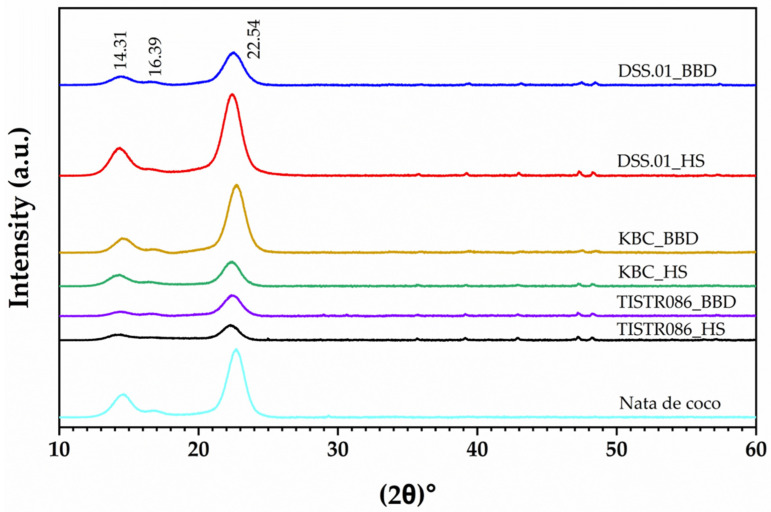
XRD of BC from standard (HS) and optimised agro-industrial waste medium (BBD). Intensity is presented in a.u. (arbitrary units).

X-ray diffraction analysis was conducted to assess the crystallinity of the BC samples, as shown in Figure 6. The Crystallinity Index (CI) was determined by evaluating the intensity of diffraction peaks observed at 2θ = 22.5° and 18.5°, which correspond to the (110) and (200) planes of cellulose I [22]. In this study, the position of *I*_002_ was around 22.5, and *I*_am_ was located at around 16.5°. The CI calculation is referenced in Equation 4. The intensity of diffraction peaks varied among the culture medium, indicating differences in their crystalline structures. However, using an agro-industrial waste medium did not significantly affect the crystallinity of the BC. The CI of nata de coco was measured at 81.99%. In comparison, the CIs for BC from *L. brevis* DSS.01, *A. tropicalis* KBC, and *K. xylinus* TISTR 086 cultivated in agro-industrial waste medium were 81.95%, 82.11%, and 81.97%, respectively. These values are comparable to those from the HS medium, which were 82.11%, 82.05%, and 82.07%. A highly ordered and crystalline structure contributes to the mechanical strength and stability of BC, while lower crystallinity may enhance its swelling properties [44,45].

### 3.6. Cost Analysis of Culture Medium Input

A simplified cost analysis was performed based on the input resources for the culture medium. the cost of culture medium input. Table 4 showed a significant cost saving when an optimised agro-industrial waste medium was applied for BC cultivation. For *L. brevis* DSS.01, the expenditure to produce 1.56 ± 0.15 g/L of BC dry weight in the optimised agro-industrial waste medium was approximately AUD 0.81. It was determined to be AUD 0.52/gram of BC dry weight. Meanwhile, the cost of producing 0.93 g/L of BC dry weight from the HS medium was AUD 4.67, which equals AUD 5.02/gram of BC dry weight. The literature reports that the major cost in BC production is the culture medium, which accounts for approximately 30% of the total production cost [46]. It could be suggested that using the optimised medium significantly reduced the total BC production cost. The presented calculation represents a preliminary, simplified estimate intended only to compare substrates under controlled laboratory conditions, and a complete techno-economic analysis (TEA) would be required for industrial-scale evaluation.

## 4. Discussion

### 4.1. Initial Medium Screening Prior to RSM Optimisation

Culture medium pre-optimisation using the OFAT method revealed the impact of cellulose-producing bacteria, carbon and nitrogen sources, and the concentrations of carbon (glucose), nitrogen (yeast extract), and MgSO_4,_ on the BC yield (Figure 1A–F). The superior BC production by *L. brevis* DSS.01 compared to traditional cellulose-producing bacteria, acetic acid bacteria (AAB), is particularly noteworthy. As it challenges the conventional view that AAB are the most efficient BC producers. This finding aligns with a previous study that demonstrated that L. plantarum could produce higher BC yields than cellulose-producing AAB under optimised conditions [19]. The variation in BC production among different strains likely reflects differences in cellulose synthase activity, metabolic pathways, or regulatory mechanisms governing cellulose biosynthesis [47,48,49].

This study supported the statement that carbon and nitrogen sources, including MgSO_4_, are critical parameters that affect BC production. The BC production in growth medium supplemented with different carbon sources indicates a diverse metabolic potential depending on the cellulose-producing bacteria used [50]. Among carbon sources used in this study, fructose, glucose, and mannitol are best for BC production for *L. brevis* DSS.01, *A. tropicalis* KBC, and *K. xylinus* TISTR 086, respectively (Figure 1B). These findings align with previous reports, which highlight strain-specific carbon utilisation patterns. Although glucose is widely recognised as a primary carbon source for BC production [51]. The isolated *L. brevis* DSS.01 may efficiently express phosphoglucose isomerase, enabling the conversion of fructose to glucose-6-phosphate before it enters the cellulose biosynthesis pathway [52]. Moreover, fructose can be metabolised via the pentose phosphate or gluconeogenesis pathways, facilitating the generation of UDP-glucose required for BC synthesis [53].

On the other hand, *K. xylinus* TISTR 086 may produce higher levels of mannitol dehydrogenase than the tested strains, allowing mannitol to serve as a more effective intermediate for UDP-glucose formation [54,55]. *Komagataeibacter* and *Acetobacter* typically lack the enzyme β-galactosidase, which hydrolyses lactose into glucose and galactose. As a result, no glucose is available for BC biosynthesis [56]. Interestingly, *L. brevis* DSS.01 was also unable to synthesise BC from lactose, as lactose is a disaccharide that requires additional metabolic steps before it becomes available, which may lead *Lactobacillus* to favour lactic acid production over BC generation. This observation also aligned with the low BC production observed for the disaccharide sucrose [56]. BC-producing strains typically lack enzymes to hydrolyse starch [32].

Nitrogen sources play a critical role in BC biosynthesis by influencing the balance between energy consumption for cell growth and for BC synthesis [55]. Yeast extract was identified as the best nitrogen source in this study (Figure 1C), due to no toxicity and ionic strength generation unlike inorganic nitrogen sources, which is in line with the literature [19,55]. Yeast extract was more effective in supporting BC production by *A. senegalensis* MA1 compared to peptone and other organic nitrogen sources [24]. Organic nitrogen is a pre-digested substrate that reduces the energy required for assimilation. This shift enables greater metabolic flux to be directed toward cellulose synthesis rather than nitrogen processing [57,58].

The BC production had a positive relationship with glucose and yeast extract concentration (Figure 1D,E). The combination of glucose and yeast extract in optimised medium has been shown to significantly increase BC yields. The BC yield of *G. persimmonis* increased with higher concentrations of peptone and yeast extract, while it decreased with lower concentrations of glucose and peptone [13]. Similarly, a high concentration of 20–25 g/L of glucose was found optimal for *Medusomyces gisevii*, yielding significant BC production [59]. However, excessive carbon and nitrogen can inhibit BC production [21,60,61]. In terms of yeast extract concentrations, yeast extract over 1.0%(*w*/*v*) was excessive for *L. brevis* DSS.01 (Figure 1E), the excess nitrogen increased biomass production and decreased BC synthesis [21,22]. It was recommended to use yeast extract combined with other nitrogen sources to improve BC production [55]. In this study, MgSO_4_ supported that the inclusion of MgSO_4_ in the culture medium positively influences BC yield and quality, especially for AAB (Figure 1F). MgSO_4_ is a source of magnesium, which is essential for enzymatic activities involved in BC biosynthesis. It presented a synergistic effect when applied with other nutrients, such as yeast extract and carbon sources, to maximise BC yields [19]. Like glucose and yeast extract, specific concentrations of MgSO_4_ can significantly boost BC production. The effect of MgSO_4_ depends on the presence of other nutrients, such as carbon and nitrogen sources, which influence the overall BC production efficiency [55]. Excessive MgSO_4_ can lead to osmotic stress, affecting BC production and altering the physicochemical properties of BC [40,41].

Whole-genome sequencing and comprehensive functional annotation of *L. brevis* DSS.01 would provide more critical insights into the genetic basis of cellulose biosynthesis in LAB. Identification and characterisation of cellulose synthase operons, regulatory elements, and associated export machinery would enable rational metabolic engineering strategies. LAB are known producers of diverse exopolysaccharides, and understanding potential dual polysaccharide production capacity would be essential for optimising BC yield. Genome-scale metabolic modelling combined with ^13^C-labeled substrate tracing could quantify carbon flux partitioning between competing biosynthetic pathways (e.g., exopolysaccharide versus bacterial cellulose production). Genetic knockout studies targeting putative cellulose synthase and exopolysaccharide biosynthesis genes would definitively establish their roles and determine relative contributions to total polysaccharide yield. Such molecular investigations would identify metabolic bottlenecks and engineering targets to enhance BC production in food-grade LAB.

### 4.2. Valorisation of Agro-Industrial Wastes for Culture Medium

Three tested cellulose-producing bacteria produced higher BC yield in PP than other alternative carbon sources (Figure 2A). The results aligned with the literature; the BC yield of mixed cultures of *K. rhaeticus* M12 and *K. intermedius* 6-5 cultivated in the optimised PP medium containing glucose 3.58% and citric acid 0.45% was 46.15% higher than that obtained from the traditional medium [2]. After optimising the enzymatically hydrolysed prickly pear peel extract through a central composite design, the BC yield of *L. plantarum* AS.6 increased significantly [62]. Hence, PP is a promising carbon source for BC production, as it retains a significant amount of sugar. RB can be used as a nitrogen source for BC production (Figure 2B). However, the untreated RB contains nutrients that are inaccessible owing to its lignocellulosic networks [22]. High concentrations of untreated RB may increase medium viscosity, leading to insoluble solids that restrict oxygen transfer and affect BC fabrication [63]. Significantly, BC production can be influenced by the intrinsic chemical composition, polyphenols, and other antioxidant compounds of the agro-industrial residues, particularly their carbohydrate and free sugar content [35]. Ideally, a complete chemical characterisation of each agro-industrial waste followed by comparison at equivalent carbon concentrations would provide deeper mechanistic insight. Future work should include complete chemical profiling and carbon-normalised comparisons to better understand how substrate composition influences BC productivity.

### 4.3. Box–Behnken Design for Culture Medium Optimisation

Across all three BC producers, the BBD results indicated that BC production was predominantly influenced by the individual independent variables and the associated curvature, rather than by pairwise synergistic or antagonistic interactions. Biologically, a high dominance of PP over others is expected because BC biosynthesis predominantly depends on the availability of carbon sources. Once sufficient carbon is provided, variations in RB or MgSO_4_ yield only minor effects on production levels. The results aligned with the literature, which stated that the availability and quality of carbon sources in the culture medium are the main factors affecting BC yield. Some nutritional sources function as supporting bacterial growth without a direct impact on BC biosynthesis [1,24,64]. Particularly, MgSO_4_ serves primarily as a mineral cofactor, and its influence tends to plateau once the cells’ basic requirements are satisfied, reducing the likelihood of simultaneous limitations imposed by carbon or complex nutrients [55].

The BBD was applied using narrow experimental ranges of PP, RB, and MgSO_4_ levels, rather than setting the experimental level toward extreme combinations that might generate unstable or unpredictable interactions. The BBD can effectively identify significant variables and capture curvature in the response. However, its layout can make interaction terms harder to detect when the experimental ranges are relatively tight, and the response surface behaves smoothly [11,65]. For these reasons, synergistic effects cannot be entirely identified. The high precision of the quadratic model and its strong fit indicate that any remaining interactions are weak within the operational window explored.

The recent study suggested that under a proper optimisation through BBD, the untreated agro-industrial waste together with MgSO_4_ can enhance BC production (Table 2 and Figure 3), which saves the cost of hydrolysis processes. Typically, agro-industrial wastes are often hydrolysed through acid or enzymatic methods, to convert complex carbohydrates into simpler sugars, prior used for bacterial fermentation [22,66]. The close match between predicted and experimental responses suggested that the conditions tested were well-suited for BC production.

The optimal condition for 10% *L. brevis* DSS.01 (expressed as % *w*/*v*) is 3.6% PP, 6.8% RB, 0.27% Na_2_HPO_4_, 0.115% citric acid, and 0.05% MgSO_4_ at pH 6. The cultures were then incubated at 28 °C for 14 days under static conditions. A previous study also reported that untreated agro-industrial waste can potentially be used for BC production through RSM. BC yield from maple syrup was comparable to that from pure fructose, suggesting that the sugar profile and trace nutrients in maple syrup may enhance bacterial metabolism. Moreover, natural components may provide additional micronutrients that support bacterial growth and BC biosynthesis [67]. Several studies reported that RSM is a reliable method for predicting the optimum conditions for BC production [14,19,24]. Optimising the culture medium composition and cultivation conditions of *A. senegalensis* MA1 using RSM led to BC yields approximately 20% higher than those from the unoptimised HS medium [24]. Similarly, BC production from pineapple peel waste increased by up to 43% through RSM [14]. The isolated *L. plantarum* produced BC 67% higher than that achieved with modified Yamanaka medium when cultivated in the optimised yeast extract, MgSO_4_, and pH in the culture medium using BBD [19].

### 4.4. Evaluation of Bacterial Cellulose Properties

BC had a high-water content ranging from 92.26% to 99.94%. Higher water content generally corresponds to greater water-holding capacity. The water content of materials also corresponds to their crystallinity, tensile strength, and degree of polymerisation [68].

The uniform fibre diameter of *L. brevis* DSS.01 BC across different media probably reflects the physical constraints imposed by the thick peptidoglycan cell wall characteristic of Gram-positive bacteria (20–80 nm thickness). This structural feature can probably distinguish Gram-positive BC producers from Gram-negative AAB, where thinner cell walls (7–8 nm peptidoglycan) allow greater fibre diameter variability (20–100 nm) in response to environmental conditions [69]. The thinner BC fibres may offer advantages for specific applications, such as improved mechanical properties, higher surface area, and enhanced water retention capacity [70,71]. The differences in *K. xylinus* strains (TISTR 086, 428, 975, 1011, and KX) resulted in the production of BC with a variation in fibre diameters and crystallinity profiles, even when grown under agitated conditions [72].

FTIR and XRD analyses confirmed the composition of a cellulose I structure in BC from this study. The high Crystallinity Index of the DSS.01_HS sample is potentially a characteristic of Gram-positive cellulose-producing bacteria. Gram-positive bacteria lack the periplasmic space and outer membrane present in Gram-negative systems, enabling direct cellulose export from the cytoplasmic membrane through the peptidoglycan layer to the extracellular environment [69]. This streamlined pathway probably minimises disruption to cellulose chain alignment, resulting in higher crystallinity (typically 90–96%) compared to Gram-negative BC (84–90%). The thick peptidoglycan cell wall (20–80 nm) may additionally act as a molecular template, enforcing parallel fibril alignment during extrusion [73]. The slight reduction in CI observed with agro-industrial waste medium likely reflects trace plant polysaccharides from substrates, though values still remain exceptionally high.

Advanced structural characterisation, including solid-state ^13^C-NMR spectroscopy, methylation linkage analysis, and enzymatic specificity testing (cellulase degradation kinetics, Congo Red binding assays), would provide definitive confirmation of cellulose structure and distinguish it from structurally similar exopolysaccharides that lactic acid bacteria commonly produce. Comparative mechanical testing (tensile strength, Young’s modulus, elongation at break, water retention capacity) of DSS.01-derived BC versus traditional AAB-derived BC would determine whether Gram-positive BC offers superior or distinct functional properties for specific applications. Investigating the impact of narrower fibre-diameter uniformity and elevated crystallinity on composite material performance may reveal niche applications where LAB-derived BC offers unique advantages over conventional BC products.

### 4.5. Culture Medium Cost Analysis

It is important to note that this study demonstrates substantial cost reduction in culture medium components through agro-waste substitution, a complete techno-economic analysis must incorporate all production costs including: (1) agro-waste acquisition, transportation, and preprocessing (drying, grinding, sterilisation), (2) fermentation equipment and operational costs (energy, labour, quality control), (3) downstream processing (purification, drying, packaging), and (4) waste disposal. Life cycle assessment should evaluate environmental impacts including carbon footprint, water usage, and waste generation relative to conventional BC production systems. Market analysis comparing the value proposition of LAB-derived BC (food-grade, GRAS status) versus traditional AAB-derived BC for various applications (food packaging, biomedical materials, composite reinforcement) will determine commercial viability. Pilot-scale validation (100–1000 L) is necessary to identify scale-up challenges, assess production consistency, and refine cost projections based on realistic industrial scenarios. Such comprehensive economic and environmental analyses are essential for attracting industrial investment and guiding commercialisation strategies.

## 5. Conclusions

The successful utilisation of agro-industrial wastes as alternative substrates for bacterial cellulose production was demonstrated. The key findings include screening various agro-industrial wastes, which revealed that pear pomace and rice bran were the most promising substitutes for carbon and nitrogen sources in the standard HS medium. Optimisation of the agro-industrial waste medium composition using Box–Behnken Design resulted in a significant increase in cellulose yield, reaching levels comparable to those in the HS medium. The optimal condition for 10% *L. brevis* DSS.01 (expressed as % *w*/*v*) is 3.6% pear pomace, 6.8% rice bran, 0.27% Na_2_HPO_4_, 0.115% citric acid, and 0.05% MgSO_4_ at pH 6. The cultures were then incubated at 28 °C for 14 days under static conditions. BC produced from the optimised agro-industrial waste medium exhibited distinct morphological, structural characteristics, and crystallinity compared to that from the HS medium, as evidenced by SEM, FTIR, and XRD analyses. The novel LAB strain, *L. brevis* DSS.01, demonstrated a unique ability to produce BC fibres with relatively high uniform diameters in response to different agro-waste substitutions, highlighting its potential for tailoring the material properties of BC for specific applications. A cost analysis indicated substantial savings in production costs when using the optimised agro-waste media. This cost reduction, coupled with the comparable BC yields obtained from the novel LAB and AAB strains, underscores the economic feasibility and sustainability of utilising agro-waste substrates for BC production. A limitation of this study is that experimental validation of the optimised agro-industrial waste medium was performed exclusively for *L. brevis* DSS.01. This prioritisation reflects the study’s focus on establishing LAB as alternative BC producers with potential food-grade applications. The comparable model fit statistics across all three strains (R^2^ > 0.95) suggest similar predictive reliability; however, future studies should validate the optimal conditions for acetic acid bacteria A. tropicalis KBC and *K. xylinus* TISTR 086 to enable direct comparison of BC production efficiency between LAB and AAB using agro-industrial waste substrates.

## Figures and Tables

**Table 1:** Experimental levels of independent variables.

Independent Variables	Actual Code	Experimental Levels		
		−1	0	+1
PP (g/L)	A	12.0	24.0	36.0
RB (g/L)	B	34.0	68.0	102.0
MgSO_4_ (g/L)	C	0.5	1.0	1.5

**Table 2:** Box–Behnken Design for the responses of bacterial cellulose production.

Trial	Experimental Level			Responses (Bacterial Cellulose Production)					
				*L. brevis* DSS.01		*A. tropicalis* KBC		*K. xylinus* TISTR 086	
	A: PP (g/L)	B: RB (g/L)	C: MgSO_4_ (g/L)	Actual (g/L)	Predicted (g/L)	Actual (g/L)	Predicted (g/L)	Actual (g/L)	Predicted (g/L)
1	12	34	1	0.46 ± 0.04	0.42	0.65 ± 0.01	0.66	0.14 ± 0.03	0.15
2	36	34	1	1.41 ± 0.10	1.36	1.23 ± 0.13	1.29	0.29 ± 0.03	0.30
3	12	102	1	0.53 ± 0.06	0.58	0.78 ± 0.05	0.73	0.25 ± 0.02	0.24
4	36	102	1	1.46 ± 0.12	1.50	1.48 ± 0.02	1.47	0.34 ± 0.03	0.33
5	12	68	0.5	0.74 ± 0.14	0.74	0.70 ± 0.08	0.72	0.22 ± 0.03	0.23
6	36	68	0.5	1.64 ± 0.08	1.65	1.45 ± 0.06	1.42	0.33 ± 0.03	0.34
7	12	68	1.5	0.55 ± 0.05	0.55	0.72 ± 0.09	0.75	0.24 ± 0.02	0.23
8	36	68	1.5	1.50 ± 0.02	1.50	1.45 ± 0.04	1.43	0.36 ± 0.05	0.35
9	24	34	0.5	1.07 ± 0.29	1.11	1.12 ± 0.1	1.09	0.14 ± 0.03	0.13
10	24	102	0.5	1.27 ± 0.13	1.22	1.20 ± 0.2	1.23	0.19 ± 0.06	0.19
11	24	34	1.5	0.85 ± 0.03	0.90	1.16 ± 0.03	1.12	0.14 ± 0.02	0.14
12	24	102	1.5	1.15 ± 0.06	1.10	1.21 ± 0.15	1.24	0.19 ± 0.01	0.20
13	24	68	1	1.29 ± 0.12	1.33	1.04 ± 0.04	0.95	0.29 ± 0.01	0.29
14	24	68	1	1.35 ± 0.08	1.33	0.96 ± 0.06	0.95	0.29 ± 0.03	0.29
15	24	68	1	1.35 ± 0.06	1.33	0.95 ± 0.09	0.95	0.24 ± 0.03	0.29
16	24	68	1	1.37 ± 0.08	1.33	0.95 ± 0.05	0.95	0.29 ± 0.02	0.29
17	24	68	1	1.30 ± 0.16	1.33	0.86 ± 0.07	0.95	0.32 ± 0.03	0.29

**Table 3:** ANOVA for the quadratic model and regression analysis of BC production using Box–Behnken Design.

	Source	Sum of Squares	Df	Mean Square	F-Value	*p*-Value
*L. brevis* DSS.01	Model	2.18	9	0.2418	71.87	<0.0001
	A-PP	1.75	1	1.75	518.78	<0.0001
	B-RB	0.0469	1	0.0469	13.93	0.0073
	C-MgSO_4_	0.0553	1	0.0553	16.45	0.0048
	AB	0.0001	1	0.0001	0.0367	0.8535
	AC	0.0005	1	0.0005	0.155	0.7055
	BC	0.002	1	0.002	0.587	0.4686
	A^2^	0.1265	1	0.1265	37.58	0.0005
	B^2^	0.1615	1	0.1615	47.99	0.0002
	C^2^	0.0113	1	0.0113	3.34	0.1102
	Residual	0.0236	7	0.0034		
	Lack of Fit	0.0192	3	0.0064	5.91	0.0595
	Pure Error	0.0043	4	0.0011		
	Cor Total	2.2	16			
	R^2^ = 0.9893, Adjusted R^2^ = 0.9755, Adequate Precision = 27.6971					
*A. tropicalis* KBC	Model	1.1	9	0.1222	29.76	<0.0001
	A-PP	0.9508	1	0.9508	231.64	<0.0001
	B-RB	0.0331	1	0.0331	8.07	0.025
	C-MgSO_4_	0.0006	1	0.0006	0.1508	0.7093
	AB	0.0034	1	0.0034	0.8378	0.3905
	AC	0.0001	1	0.0001	0.0157	0.9038
	BC	0.0002	1	0.0002	0.0449	0.8382
	A^2^	0	1	0	0.0026	0.9604
	B^2^	0.0314	1	0.0314	7.65	0.0279
	C^2^	0.0741	1	0.0741	18.05	0.0038
	Residual	0.0287	7	0.0041		
	Lack of Fit	0.0129	3	0.0043	1.08	0.451
	Pure Error	0.0158	4	0.004		
	Cor Total	1.13	16			
	R^2^ = 0.9745, Adjusted R^2^ = 0.9418, Adequate Precision = 16.651					
*K. xylinus* TISTR 086	Model	0.0785	9	0.0087	15.89	0.0007
	A-PP	0.0276	1	0.0276	50.22	0.0002
	B-RB	0.0079	1	0.0079	14.33	0.0068
	C-MgSO_4_	0.0002	1	0.0002	0.4153	0.5398
	AB	0.001	1	0.001	1.84	0.2165
	AC	0	1	0	0.0421	0.8432
	BC	2.39 × 10^−6^	1	2.39 × 10^−6^	0.0043	0.9493
	A^2^	0.009	1	0.009	16.48	0.0048
	B^2^	0.0248	1	0.0248	45.21	0.0003
	C^2^	0.0087	1	0.0087	15.86	0.0053
	Residual	0.0038	7	0.0005		
	Lack of Fit	0.0007	3	0.0002	0.2865	0.8338
	Pure Error	0.0032	4	0.0008		
	Cor Total	0.0823	16			
	R^2^ = 0.9533, Adjusted R^2^ = 0.8933, Adequate Precision = 12.5485					

**Table 4:** Cost analysis of culture medium input for bacterial cellulose produced, comparing HS medium with optimised agro-industrial waste medium.

Medium Composition	Unit	Price		HS (1 L)		Optimised BBD (1 L)	
		AUD/unit	AUD/g	Quantity (g)	Cost	Quantity (g)	Cost
Glucose	500 g	14.25	0.03	20.00	0.57	0	0
Yeast extract	500 g	103.00	0.21	5.00	1.03	0	0
Peptone	500 g	284.92	0.57	5.00	2.85	0	0
Na_2_HPO_4_	500 g	24.35	0.05	2.70	0.13	2.70	0.13
Citric acid	500 g	18.95	0.04	1.15	0.04	1.15	0.04
MgSO_4_	500 g	21.55	0.04	1.00	0.04	0.50	0.02
PP	1000 g	0.30	0.0003	0	0	36.00	0.01
RB	15 kg	90.00	0.006	0	0	102.00	0.61
Total cost					4.67		0.81

## Data Availability

The raw data supporting the conclusions of this article will be made available by the authors on request. The 16S rRNA data for the molecular identification of the isolate is available at the NCBI as Genbank PX578036.1.

## Supplementary Materials

The following supporting information can be downloaded at: https://www.mdpi.com/article/10.3390/foods15020394/s1, Figure S1: Agarose gel electrophoresis of amplified DNA fragments of reference bacteria compared with the isolated strains: (A) TISTR086, (B) KBC, and (C) DSS.01; Figure S2: Neighbour-joining phylogenetic tree: (A) KBC and (B) DSS.01; Figure S3: Original SEM micrographs of BC; Figure S4: Fibre size distribution of BC; Figure S5: Culture medium optimisation for BC production: (A) experimental setup for screening of agro-industrial wastes, (B) experimental setup for Box–Behnken Design, (C) model validation of the predicted optimal conditions for *L. brevis* DSS.01, (D) BC pellicles before and after purification of the 2 L upscale production batch; Table S1: Colony morphology and Gram staining of suspected cellulose-producing strains; Table S2: 16S rDNA sequencing analysis of suspect cellulose-producing bacteria; Table S3: Culture medium substitution for a screening of agro-industrial wastes; Table S4: Calculation of pear pomace and rice bran at a centre level of BBD (level 0); Table S5: Water content of bacterial cellulose samples.

## Abbreviations

The following abbreviations are used in this manuscript:

AAB	Acetic acid bacteria
ANOVA	Analysis of Variance
AP	Apple pomaces
BBD	Box–Behnken Design
BC	Bacterial cellulose
BP	Beetroot pomace
CI	Crystallinity Index
DSS.01_BBD	Bacterial cellulose from *L. brevis* DSS.01 cultivated in optimal agro-industrial waste medium
DSS.01_HS	Bacterial cellulose from *L. brevis* DSS.01 cultivated in HS medium
FTIR	Fourier transform infrared spectroscopy
HS	Hestrin-Schramm
*I*_110_	Maximum intensity of the XRD peaks
*I*_am_	Minimum intensity of the XRD peaks
KBC_BBD	Bacterial cellulose from *A. tropicalis* KBC cultivated in optimal agro-industrial waste medium
KBC_HS	Bacterial cellulose from *A. tropicalis* KBC cultivated in HS medium
LAB	Lactic acid bacteria
O.D.	Optical Density
OFAT	One-factor-at-a-time
PP	Pear pomaces
RB	Rice bran
rpm	Revolutions Per Minute
RSM	Response surface methodology
SCOBY	Symbiotic culture of bacteria and yeast
SEM	Scanning electron microscopy
s/step	Second per step
TISTR	Thailand Institute of Scientific and Technological Research
TISTR086_BBD	Bacterial cellulose from *K. xylinus* TISTR086 cultivated in optimal agro-industrial waste medium
TISTR086_HS	Bacterial cellulose from *K. xylinus* TISTR086 cultivated in HS medium
WBS	Waste beer slurry
*W*_dry_	Dry weight
*W*_wet_	Wet weight
XRD	X-ray diffraction

## References

[1] Ma X., Yuan H., Wang H., Yu H. (2021). Coproduction of bacterial cellulose and pear vinegar by fermentation of pear peel and pomace. Bioprocess Biosyst. Eng..

[2] El-Gendi H., Taha T.H., Ray J.B., Saleh A.K. (2022). Recent advances in bacterial cellulose: A low-cost effective production media, optimization strategies and applications. Cellulose.

[3] Jaroennonthasit W., Lam N.T., Sukyai P. (2021). Evaluation of carbon sources from sugar industry to bacterial nanocellulose produced by *Komagataeibacter xylinus*. Int. J. Biol. Macromol..

[4] Hungund B., Prabhu S., Shetty C., Acharya S., Prabhu V., Gupta S.G. (2013). Production of bacterial cellulose from *Gluconacetobacter persimmonis* GH-2 using dual and cheaper carbon sources. J. Microb. Biochem. Technol..

[5] Patel A., Patel P., Shukla A., Wong J.W.C., Varjani S., Gosai H. (2023). Sustainable bioconversion of industrial wastes into bacterial cellulose for diverse applications: A way towards pollution control and abatement. Curr. Pollut. Rep..

[6] Xu Y., Wei K., Bian L., Li G., Zhang C. (2025). High-yield bacterial cellulose production from rice bran using a genetically characterized *Komagataeibacter europaeus* strain. Int. J. Biol. Macromol..

[7] Fight Food Waste CRC Options for Utilising Apple and Pear Pulp Residue. https://endfoodwaste.com.au/options-for-utilising-apple-and-pear-pulp-residue/.

[8] United States Department of Agriculture Australia Rice Area, Yield and Production, International Production Assessment Division. https://ipad.fas.usda.gov/countrysummary/Default.aspx?id=AS&crop=Rice.

[9] Peanparkdee M., Iwamoto S. (2019). Bioactive compounds from by-products of rice cultivation and rice processing: Extraction and application in the food and pharmaceutical industries. Trends Food Sci. Technol..

[10] Box G.E.P., Behnken D.W. (1960). Some new three level designs for the study of quantitative variables. Technometrics.

[11] Nair A.T., Makwana A.R., Ahammed M. (2014). The use of response surface methodology for modelling and analysis of water and wastewater treatment processes: A review. Water Sci. Technol..

[12] Bae S., Shoda M. (2005). Statistical optimization of culture conditions for bacterial cellulose production using Box-Behnken design. Biotechnol. Bioeng..

[13] Hegde S., Bhadri G., Narsapur K., Koppal S., Oswal P., Turmuri N., Jumnal V., Hungund B. (2013). Statistical optimization of medium components by response surface methodology for enhanced production of bacterial cellulose by *Gluconacetobacter persimmonis*. J. Bioprocess Biotech..

[14] Patel A., Patel P., Parmar M., Gosai H. (2024). Employing RSM and ANN-based applications for modelling enhanced bacterial cellulose production from pineapple peel waste using *Komagateibacter saccharivorans* APPK1. Chem. Eng. J..

[15] Hao Z., Zhang W., Wang X., Wang Y., Qin X., Luo H., Huang H., Su X. (2022). Identification of WxL and S-layer proteins from *Lactobacillus brevis* with the ability to bind cellulose and xylan. Int. J. Mol. Sci..

[16] Adetunji V.O., Adegoke G.O. (2007). Bacteriocin and cellulose production by lactic acid bacteria isolated from West African soft cheese. Afr. J. Biotechnol..

[17] Olawoye B., Jolayemi O.S., Origbemisoye B.A., Oluwajuyitan T.D., Popoola-Akinola O. (2023). Hydrolysis of starch. Starch: Advances in Modifications.

[18] Gunkova P.I., Buchilina A.S., Maksimiuk N.N., Bazarnova Y.G., Girel K.S. (2021). Carbohydrate fermentation test of lactic acid starter cultures. IOP Conf. Ser. Earth Environ. Sci..

[19] Saleh A.K., El-Gendi H., Soliman N.A., El-Zawawy W.K., Abdel-Fattah Y.R. (2022). Bioprocess development for bacterial cellulose biosynthesis by novel *Lactiplantibacillus plantarum* isolate along with characterization and antimicrobial assessment of fabricated membrane. Sci. Rep..

[20] Digel I., Akimbekov N., Rogachev E., Pogorelova N. (2023). Bacterial cellulose produced by *Medusomyces gisevii* on glucose and sucrose: Biosynthesis and structural properties. Cellulose.

[21] Rani M.U., Appaiah A. (2011). Optimization of culture conditions for bacterial cellulose production from *Gluconacetobacter hansenii* UAC09. Ann. Microbiol..

[22] Henry S., Dhital S., Sumer H., Butardo J. (2024). Solid-state fermentation of cereal waste improves the bioavailability and yield of bacterial cellulose production by a *Novacetimonas* sp. Isolate. Foods.

[23] Yilmaz M., Goksungur Y. (2024). Optimization of bacterial cellulose production from waste figs by *Komagataeibacter xylinus*. Fermentation.

[24] Aswini K., Gopal N.O., Uthandi S. (2020). Optimized culture conditions for bacterial cellulose production by *Acetobacter senegalensis* MA1. BMC Biotechnol..

[25] Margaretty E., Dewi E., Kalsum L., Ningsih A.S., Amin J.M. (2021). Effect of sugar, ammonium sulfate and magnesium sulfate as supplementary nutrients in coconut water fermented by *Acetobacter xylinum* to produce biocellulose membranes. Atlantis Highlights in Engineering, Proceedings of the 4th Forum in Research, Science, and Technology, Palembang, Sumatra, 10–11 November 2020.

[26] Demishtein K., Reifen R., Shemesh M. (2019). Antimicrobial properties of magnesium open opportunities to develop healthier food. Nutrients.

[27] Skiba E.A., Gladysheva E.K., Budaeva V.V., Aleshina L.A., Sakovich G.V. (2022). Yield and quality of bacterial cellulose from agricultural waste. Cellulose.

[28] Narh C., Frimpong C., Mensah A., Wei Q. (2018). Rice bran, an alternative nitrogen source for *Acetobacter xylinum* bacterial cellulose synthesis. Bioresour. Bioprocess..

[29] Malhi N., Carragher J., Saarela M., Pahl S. (2021). A Review of Opportunities to Recover Value from Apple and Pear Pomace.

[30] Peng F., Ren X., Du B., Chen L., Yu Z., Yang Y. (2022). Structure, physicochemical property, and functional activity of dietary fiber obtained from pear fruit pomace (*Pyrus ussuriensis* Maxim) via different extraction methods. Foods.

[31] Sapwarobol S., Saphyakhajorn W., Astina J. (2021). Biological functions and activities of rice bran as a functional ingredient: A review. Nutr. Metab. Insights.

[32] Domingues S.Z., Timmers L.F.S.M., Granada C.E. (2022). Cellulase production by bacteria is a strain-specific characteristic with a high biotechnological potential. A review of cellulosome of highly studied strains. Cellulose.

[33] Matsuzaki H., Tochitani S., Watanabe T., Tsukahara T., Maehara K., Asaoka K., Shimizu R., Maehara Y., Kawase T. (2022). Fermented rice bran supplementation ameliorates obesity *via* gut microbiota and metabolism modification in female mice. J. Clin. Biochem. Nutr..

[34] Bi H., Wang Y., Guo Y., Ma Y. (2023). Effect of steam flash-explosion on physicochemical properties and structure of high-temperature denatured defatted rice bran protein isolate. Molecules.

[35] Zhang S., Winestrand S., Guo X., Chen L., Hong F., Jönsson L.J. (2014). Effects of aromatic compounds on the production of bacterial nanocellulose by *Gluconacetobacter xylinus*. Microb. Cell Factories.

[36] Chua G.K., Mahadi N.I.F., Tan F.H.Y., Venkatramanan V., Shah S., Prasad R. (2021). Bacterial cellulose production from agro-Industrial and food Wastes. Bio-Valorization of Waste.

[37] Bilgi E., Bayir E., Sendemir-Urkmez A., Hames E.E. (2016). Optimization of bacterial cellulose production by *Gluconacetobacter xylinus* using carob and haricot bean. Int. J. Biol. Macromol..

[38] Qui N.H., Linh N.T. (2025). Nutritive and therapeutic value of fermented rice bran as a feed additive for enhancing performance and health in chickens: A review. Open Vet. J..

[39] Castanho A., Lageiro M., Calhelha R.C., Ferreira I.C.F.R., Sokovic M., Cunha L.M., Brites C. (2019). Exploiting the bioactive properties of γ-oryzanol from bran of different exotic rice varieties. Food Funct..

[40] Altunordu H., Kayra N., Kalkan M., Üstün-Aytekin Ö., Nikerel E., Aytekin A.Ö. (2025). Enhancing bacterial cellulose yield and quality through controlled stress conditions in *Komagataeibacter xylinus* fermentation. Polym. Adv. Technol..

[41] Catarino R.P.F., Mascareli V.A.B., Costa V.L.L., Pavanello A.C.L., Spinosa W.A. (2025). Sustainability and influencing factors in bacterial cellulose production: A review of the impact of microorganisms, culture media and cultivation methods. Food Technol. Biotechnol..

[42] Fan M. (2012). Fourier transform infrared spectroscopy for natural fibres. Fourier Transform: Materials Analysis.

[43] Shi Z., Xu G., Deng J., Dong M., Murugadoss V., Liu C., Shao Q., Wu S., Guo Z. (2019). Structural characterization of lignin from *D. sinicus* by FTIR and NMR techniques. Green Chem. Lett. Rev..

[44] Campano C., Rivero-Buceta V., Fabra M.J., Prieto M.A. (2022). Gaining control of bacterial cellulose colonization by polyhydroxyalkanoate-producing microorganisms to develop bioplasticized ultrathin films. Int. J. Biol. Macromol..

[45] Yu K., Balasubramanian S., Pahlavani H., Mirzaali M.J., Zadpoor A.A., Aubin-Tam M.-E. (2020). Spiral honeycomb microstructured bacterial cellulose for increased strength and toughness. ACS Appl. Mater. Interfaces.

[46] Jozala A.F., de Lencastre-Novaes L.C., Lopes A.M., de Carvalho Santos-Ebinuma V., Mazzola P.G., Pessoa A., Grotto D., Gerenutti M., Chaud M.V. (2016). Bacterial nanocellulose production and application: A 10-year overview. Appl. Microbiol. Biotechnol..

[47] Ishiya K., Kosaka H., Inaoka T., Kimura K., Nakashima N. (2022). Comparative genome analysis of three *Komagataeibacter* strains used for practical production of Nata-de-coco. Front. Microbiol..

[48] Liang S. (2023). Advances in drug delivery applications of modified bacterial cellulose-based materials. Front. Bioeng. Biotechnol..

[49] Sun L., Zhang Y., Guo X., Zhang L., Zhang W., Man C., Jiang Y. (2020). Characterization and transcriptomic basis of biofilm formation by *Lactobacillus plantarum* J26 isolated from traditional fermented dairy products. Food Sci. Technol..

[50] Nascimento F.X., Torres C.A.V., Freitas F., Reis M.A.M., Crespo M.T.B. (2021). Functional and genomic characterization of *Komagataeibacter uvaceti* FXV3, a multiple stress resistant bacterium producing increased levels of cellulose. Biotechnol. Rep..

[51] Sumardee N.S.J., Mohd-Hairul A.R., Mortan S.H. (2020). Effect of inoculum size and glucose concentration for bacterial cellulose production by *Lactobacillus acidophilus*. IOP Conf. Ser. Mater. Sci. Eng..

[52] Wang S.-S., Han Y.-H., Chen J.-L., Zhang D.-C., Shi X.-X., Ye Y.-X., Chen D.-L., Li M. (2018). Insights into bacterial cellulose biosynthesis from different carbon sources and the associated biochemical transformation pathways in *Komagataeibacter* sp. W1. Polymers.

[53] Tabaii M.J., Emtiazi G. (2016). Comparison of bacterial cellulose production among different strains and fermented media. Appl. Food Biotechnol..

[54] Bielecki S., Krystynowicz A., Turkiewicz M., Kalinowska H. (2004). Bacterial cellulose. Biopolymers Online.

[55] Yodsuwan N., Owatworakit A., Ngaokla A., Tawichai N., Soykeabkaew N. Effect of carbon and nitrogen sources on bacterial cellulose production for bionanocomposite materials. Proceedings of the 1st Mae Fah Luang University International Conference 2012.

[56] Singh G., Gauba P., Mathur G., Rani V., Jain C.K., Gauba P. (2025). Exploring the biosynthesis, production, and functional properties of bacterial cellulose. Innovative Advancements in Biotechnology.

[57] Franklin O., Cambui C.A., Gruffman L., Palmroth S., Oren R., Näsholm T. (2016). The carbon bonus of organic nitrogen enhances nitrogen use efficiency of plants: The carbon bonus of organic nitrogen. Plant Cell Environ..

[58] Jeong S.W., Nam S.W., HwangBo K., Jeong W.J., Jeong B., Chang Y.K., Park Y.-I. (2017). Transcriptional regulation of cellulose biosynthesis during the early phase of nitrogen deprivation in nannochloropsis salina. Sci. Rep..

[59] Gladysheva E.K., Skiba E.A., Zolotukhin V.N., Sakovich G.V. (2018). Study of the conditions for the biosynthesis of bacterial cellulose by the producer *Medusomyces gisevii* Sa-12. Appl. Biochem. Microbiol..

[60] Lin Y.J., Sung W.P., Chen T.Y., Lin J.H., Kuo J.C. (2011). Production of bacterial cellulose by *Gluconacetobacter xylinus* using Taguchi methods. Appl. Mech. Mater..

[61] Sperotto G., Stasiak L.G., Godoi J.P.M.G., Gabiatti N.C., De Souza S.S. (2021). A review of culture media for bacterial cellulose production: Complex, chemically defined and minimal media modulations. Cellulose.

[62] El-Gendi H., Salama A., El-Fakharany E.M., Saleh A.K. (2023). Optimization of bacterial cellulose production from prickly pear peels and its *ex situ* impregnation with fruit byproducts for antimicrobial and strawberry packaging applications. Carbohydr. Polym..

[63] Kim S., Li H., Oh I., Kee C., Kim M. (2012). Effect of viscosity-inducing factors on oxygen transfer in production culture of bacterial cellulose. Korean J. Chem. Eng..

[64] Gullo M., La China S., Petroni G., Di Gregorio S., Giudici P. (2019). Exploring K2G30 genome: A high bacterial cellulose producing strain in glucose and mannitol based media. Front. Microbiol..

[65] 11.2.2 Box-Behnken Designs: Pennsylvania State University. https://online.stat.psu.edu/stat503/lesson/11/11.2/11.2.2.

[66] Kadier A., Ilyas R.A., Huzaifah M.R.M., Harihastuti N., Sapuan S.M., Harussani M.M., Azlin M.N.M., Yuliasni R., Ibrahim R., Atikah M.S.N. (2021). Use of industrial wastes as sustainable nutrient sources for bacterial cellulose (BC) production: Mechanism, advances, and future perspectives. Polymers.

[67] Zeng X., Small D.P., Wan W. (2011). Statistical optimization of culture conditions for bacterial cellulose production by *Acetobacter xylinum* BPR 2001 from maple syrup. Carbohydr. Polym..

[68] Rebelo A.R., Archer A.J., Chen X., Liu C., Yang G., Liu Y. (2018). Dehydration of bacterial cellulose and the water content effects on its viscoelastic and electrochemical properties. Sci. Technol. Adv. Mater..

[69] Silhavy T.J., Kahne D., Walker S. (2010). The bacterial cell envelope. Cold Spring Harb. Perspect. Biol..

[70] Kopač T., Ručigaj A. (2024). Impact of fiber diameter and surface substituents on the mechanical and flow properties of sonicated cellulose dispersions. Int. J. Biol. Macromol..

[71] Raman A., Jayan J.S., Deeraj B.D.S., Saritha A., Joseph K. (2021). Electrospun nanofibers as effective superhydrophobic surfaces: A brief review. Surf. Interfaces.

[72] Singhsa P., Narain R., Manuspiya H. (2018). Physical structure variations of bacterial cellulose produced by different *Komagataeibacter xylinus* strains and carbon sources in static and agitated conditions. Cellulose.

[73] Gorgieva S., Trček J. (2019). Bacterial Cellulose: Production, Modification and Perspectives in Biomedical Applications. Nanomaterials.

