# Supplementary material for: Bacterial Cellulose Production by a Novel Levilactobacillus brevis Isolate Using Response Surface-Optimised Agro-Industrial Substrates
Source: Foods. 2026 Jan 22;15(2):394. doi: 10.3390/foods15020394 (PMC12840732; doi:10.3390/foods15020394)
Supplement: Supplementary file 1 [file foods-15-00394-s001.zip › foods-4040050-supplementary.pdf]

Supplementary Materials:

Bacterial Cellulose Production by a Novel Levilactobacillus brevis Isolate Using Response Surface-Optimized Agro-industrial Substrates

Panyot Mongkolchat [1,2], François Malherb [1], Enzo Palombo [1], and Vito Butardo Jr [1,*]

[1] Department of Chemistry and Biotechnology, Swinburne University of Technology, Hawthorn, VIC 3122, Australia

[2] Department of Science Service, Ministry of Higher Education, Science Research and Innovation, Ratchathewi, Bangkok 10400, Thailand

* Correspondence: vbutardo@swin.edu.au

Supplementary Information

Supplementary Figure

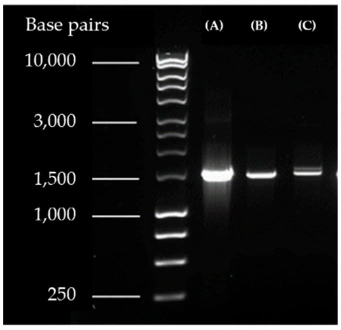

Figure S1. Agarose gel electrophoresis of amplified DNA fragments of reference bacteria compared with the isolated strains: (a) *K. xylinus* TISTR086, (b) KBC, and (c) DSS.01.

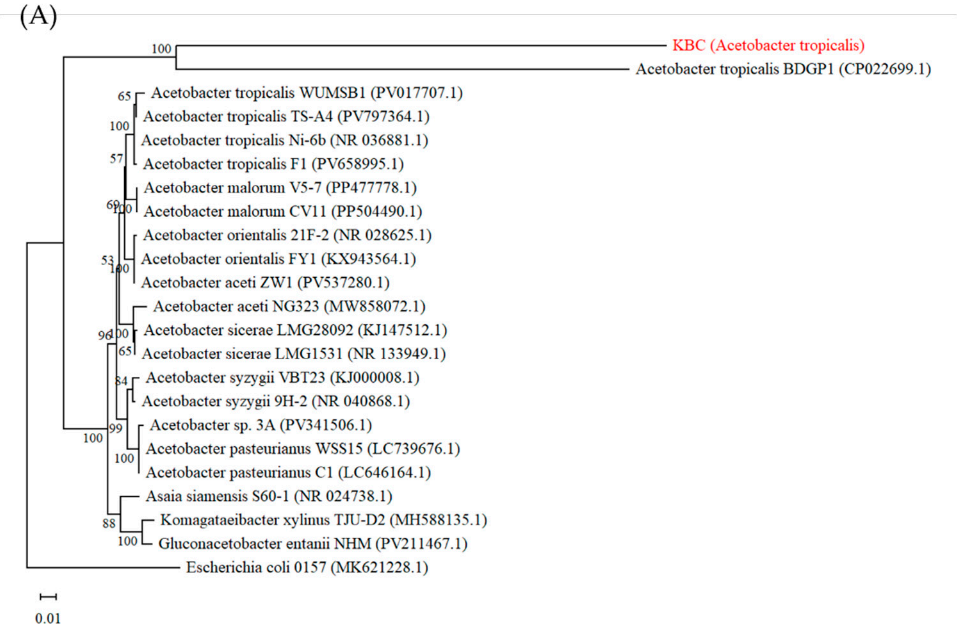

(B)

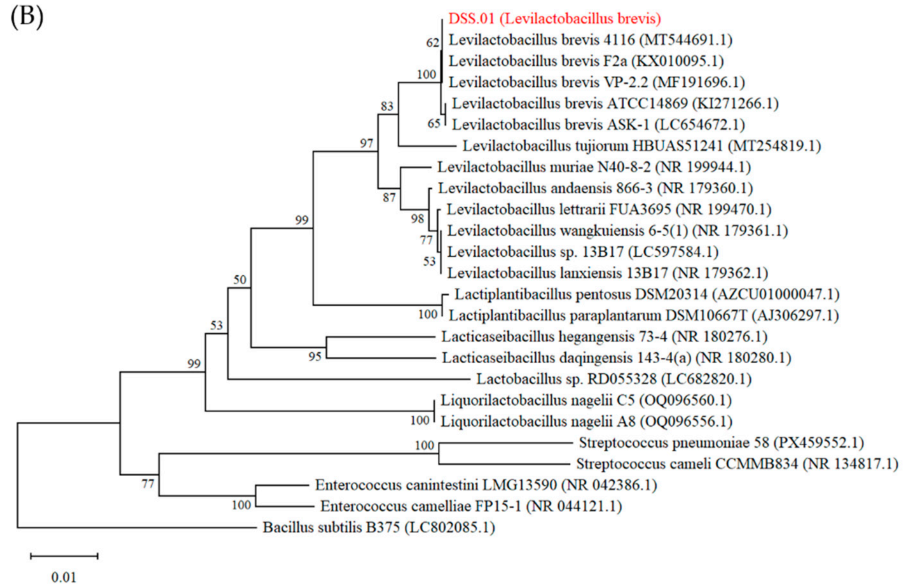

Figure S2. Neighbor-joining phylogenetic tree: (A) KBC and (B) DSS.01.

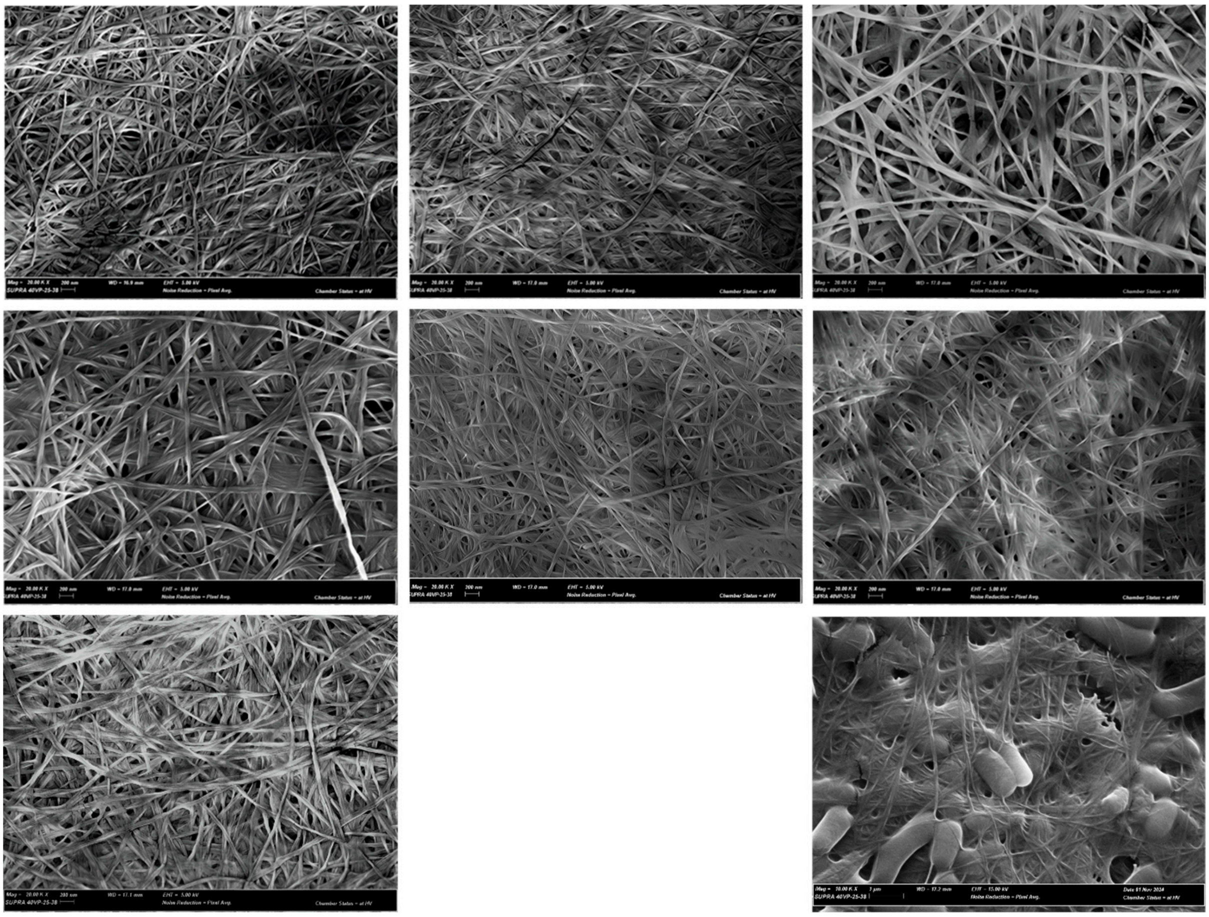

Figure S3. Original SEM micrographs of BC.

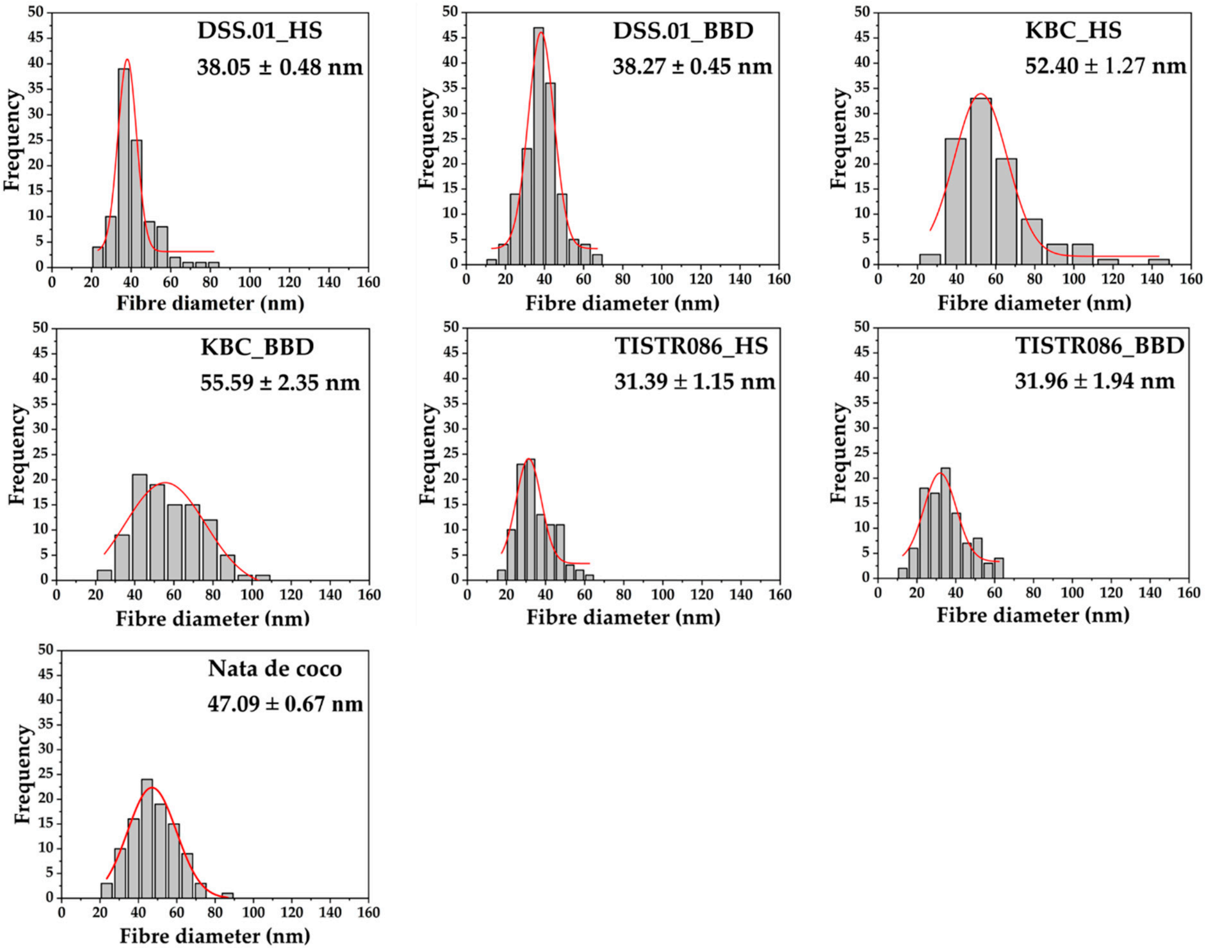

Figure S4. Fibre size distribution of BC.

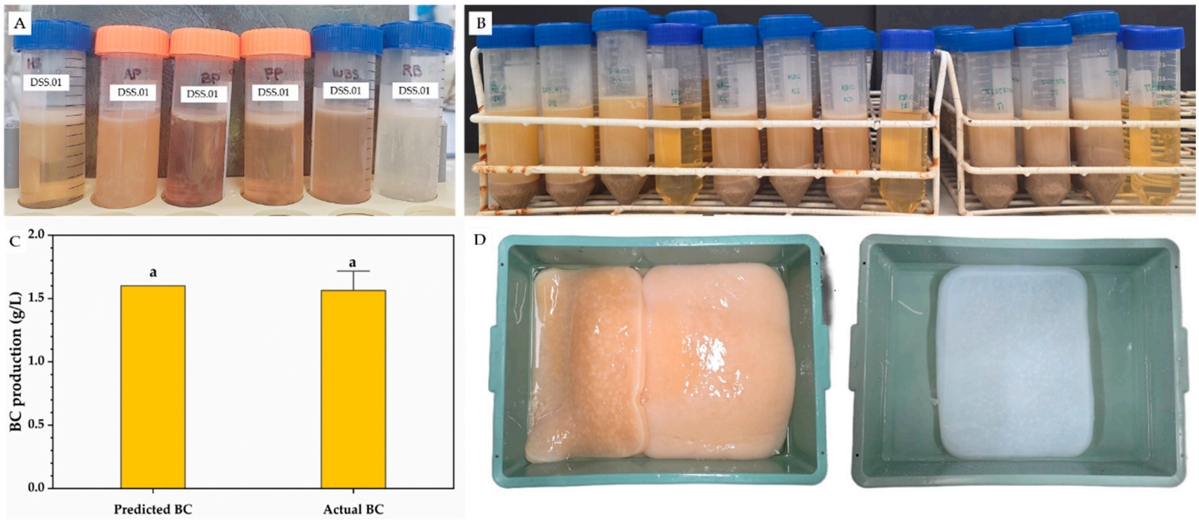

Figure S5. Culture medium optimisation for BC production: (A) experimental setup for screening of agro-industrial wastes, (B) experimental setup for Box-Behnken Design, (C) model validation of the predicted optimal conditions for *L. brevis* DSS.01, (D) BC pellicles before and after purification of the 2L upscale production batch.

Supplementary Table

Table S1. Colony morphology and Gram staining of suspected cellulose-producing strains.

Colony morphology	Characteristic	Reference AAB	AAB	LAB
		K. xylinus TISTR086	KBC	DSS.01
Configuration	Circular	+	+	+
Margin	Entire	+	+	+
Elevation	Convex	+	+	+
Surface	Mucoid	+	+	+
Opacity	Opaque	+	+	+
Pigment	Creamy white	+	+	+
Gram staining	G+/ G-	G-	G-	G+
Cell shape	Rod	+	+	+
Arrangement	Paired/ singles	Paired	Paired	Singles

Region: (+) is the present of the characteristic; (G+) is Gram-positive; (G-) is Gram-negative

Table S2. 16S rDNA sequencing analysis of suspect cellulose-producing bacteria.

Bacterial code	Description	Total score	Query cover	Identity	Accession length	Accession
KBC	*Acetobacter tropicalis*	12415	100%	99.78%	3988649	CP022699.1
	Acetobacter sicerae	2488	100%	99.71%	1426	KM485585.1
	Acetobacter senegalensis	2483	100%	99.63%	1430	MG279704.1
DSS.01	*Levilactobacillus brevis*	2590	100%	100%	1438	OP102506.1

Table S3. Culture medium substitution for a screening of agro-industrial wastes

Medium compositions (g/L)	HS medium	C substitution	N substitution
Glucose	20.0	-	20.0
Yeast extract	5.0	5.0	-
Peptone	5.0	5.0	-
Na_2HPO_4	2.7	2.7	2.7
Citric acid	1.15	1.15	1.15
AP, BP, or PP	-	20.0	-
RB or WBS	-	-	10.0

Region: (AP) apple pomaces; (BP) beetroot pomaces; (PP) pear pomace; (RB) rice bran; (WBS) waste beer slurry

The agro-industrial wastes, PP and RB, at a center level of Box-Behnken design (level 0) for culture medium optimization were calculated based on the glucose and protein content reported for those wastes. The calculation is shown in Table S5.

Table S4. Calculation of pear pomace and rice bran at a center level of BBD (level 0).

Substituents	Calculation
PP	Total carbohydrate 92.59 g x $\frac{162}{180}$ (conversion factor) = 83.3 g of glucose in 100.0 g PP<br>83.3 g of glucose is present in PP 100.0 g.<br>To replace 20.0 g of glucose in the HS medium, it needs to weigh PP = $\frac{20.0 \times 100}{83.3}$ = 24.0 g
RB	14.7 g of protein is present in RB 100.0 g.<br>To replace 10.0 g of nitrogen in the HS medium, it needs to weigh RB = $\frac{10.0 \times 100}{14.7}$ = 68.0 g<br>RB consists of considerable carbohydrates. However, the majority of carbohydrates are insoluble, primarily as dietary fiber, and are therefore not included in this calculation.

Table S5. Water content of bacterial cellulose samples.

Experiment	Medium	Water content (%)		
		DSS.01	KBC	TISTR086
Pre-optimization *Cellulose-producing bacteria*	Inoculum	99.00 ± 0.01	99.11 ± 0.03	99.08 ± 0.03
*Carbon sources**	Glucose	99.00 ± 0.02	99.15 ± 0.06	99.08 ± 0.06
	Fructose	99.17 ± 0.12	99.34 ± 0.05	99.49 ± 0.07
	Sucrose	92.35 ± 1.50	92.26 ± 0.14	99.94 ± 0.02
	Mannitol	98.69 ± 0.11	98.73 ± 0.16	99.25 ± 0.04
	Glycerol	99.72 ± 0.03	99.13 ± 0.05	99.61 ± 0.01
*Nitrogen sources**	Yeast extract	96.65 ± 0.11	94.11 ± 3.07	97.63 ± 0.79
	Peptone	96.70 ± 1.43	95.88 ± 0.71	98.17 ± 0.86
Glucose concentration	0%	99.49 ± 0.06	99.44 ± 0.78	98.74 ± 0.13
	1.0%	98.70 ± 0.03	98.98 ± 0.06	99.09 ± 0.03
	2.0%	98.38 ± 0.01	99.09 ± 0.04	98.80 ± 0.06
	3.0%	98.80 ± 0.01	99.00 ± 0.07	98.78 ± 0.07
	4.0%	98.61 ± 0.03	98.81 ± 0.03	98.94 ± 0.06
Yeast extract concentration	0%	99.81 ± 0.02	99.74 ± 0.01	99.32 ± 0.09
	0.5%	99.08 ± 0.06	99.01 ± 0.04	98.76 ± 0.01
	1.0%	98.70 ± 0.05	98.88 ± 0.04	99.05 ± 0.02
	1.5%	98.63 ± 0.04	98.81 ± 0.09	99.07 ± 0.04
	2.0%	98.94 ± 0.04	98.76 ± 0.04	98.91 ± 0.03
$MgSO_4$ concentration	0%	98.38 ± 0.02	99.09 ± 0.04	98.80 ± 0.06
	0.2%	98.38 ± 0.01	98.89 ± 0.01	98.52 ± 0.02
	0.4%	98.38 ± 0.01	98.85 ± 0.03	98.43 ± 0.06
	0.6%	98.48 ± 0.09	98.61 ± 0.04	98.38 ± 0.05
	0.8%	98.58 ± 0.04	98.62 ± 0.02	98.38 ± 0.04
Screening of agro-industrial wastes	HS	99.03 ± 0.10	99.30 ± 0.24	99.24 ± 0.45
	AP	98.84 ± 0.28	99.11 ± 0.12	99.28 ± 0.19
	BP	98.86 ± 0.54	99.26 ± 0.16	99.46 ± 0.37
	PP	98.99 ± 0.08	98.97 ± 0.22	99.26 ± 0.16
	RB	98.78 ± 0.06	98.90 ± 0.21	98.84 ± 0.39
	WBS	99.07 ± 0.56	98.92 ± 0.35	99.23 ± 0.24

Culture medium optimization	1	99.07 ± 0.03	99.01 ± 0.01	99.60 ± 0.13
	2	98.87 ± 0.05	98.56 ± 0.06	99.27 ± 0.06
	3	98.86 ± 0.04	98.41 ± 0.03	99.37 ± 0.02
	4	98.88 ± 0.03	98.52 ± 0.02	99.05 ± 0.05
	5	98.67 ± 0.14	98.85 ± 0.07	99.45 ± 0.07
	6	98.76 ± 0.05	98.34 ± 0.01	99.12 ± 0.03
	7	99.17 ± 0.05	98.92 ± 0.12	99.34 ± 0.04
	8	98.7 8± 0.01	98.52 ± 0.01	99.22 ± 0.01
	9	98.94 ± 0.08	98.64 ± 0.10	99.54 ± 0.07
	10	98.95 ± 0.04	98.72 ± 0.01	99.49 ± 0.08
	11	99.02 ± 0.03	98.69 ± 0.15	99.21 ± 0.04
	12	98.96 ± 0.04	98.54 ± 0.14	99.11 ± 0.01
	13	98.94 ± 0.05	98.64 ± 0.03	99.36 ± 0.02
	14	98.90 ± 0.05	98.45 ± 0.05	99.47 ± 0.06
	15	98.94 ± 0.03	98.28 ± 0.12	99.42 ± 0.05
	16	98.82 ± 0.02	98.67 ± 0.04	99.31 ± 0.01
	17	98.95 ± 0.10	98.67 ± 0.06	99.17 ± 0.04

* None of cellulose-producing bacterial produced BC when cultivated in a medium containing lactose, starch and inorganic nitrogen (ammonium acetate, ammonium chloride, ammonium nitrate, ammonium sulfate, and casamino acid).
